# Whole‐genome re‐sequencing reveals the impact of the interaction of copy number variants of the *rhg1* and *Rhg4* genes on broad‐based resistance to soybean cyst nematode

**DOI:** 10.1111/pbi.13086

**Published:** 2019-02-20

**Authors:** Gunvant B. Patil, Naoufal Lakhssassi, Jinrong Wan, Li Song, Zhou Zhou, Mariola Klepadlo, Tri D. Vuong, Adrian O. Stec, Sondus S. Kahil, Vincent Colantonio, Babu Valliyodan, J. Hollis Rice, Sarbottam Piya, Tarek Hewezi, Robert M. Stupar, Khalid Meksem, Henry T. Nguyen

**Affiliations:** ^1^ Division of Plant Sciences University of Missouri Columbia MO USA; ^2^ Department Agronomy and Plant Genetics University of Minnesota St. Paul MN USA; ^3^ Department of Plant, Soil and Agricultural Systems Southern Illinois University Carbondale IL USA; ^4^ Department of Plant Sciences University of Tennessee Knoxville TN USA

**Keywords:** SCN, soybean, copy number variation, haplotype analysis, *rhg1*, broad‐based resistance

## Abstract

Soybean cyst nematode (SCN) is the most devastating plant‐parasitic nematode. Most commercial soybean varieties with SCN resistance are derived from PI88788. Resistance derived from PI88788 is breaking down due to narrow genetic background and SCN population shift. PI88788 requires mainly the *rhg1‐b* locus, while ‘Peking’ requires *rhg1‐a* and *Rhg4* for SCN resistance. In the present study, whole genome re‐sequencing of 106 soybean lines was used to define the *Rhg* haplotypes and investigate their responses to the SCN HG‐Types. The analysis showed a comprehensive profile of SNPs and copy number variations (CNV) at these loci. CNV of *rhg1* (GmSNAP18) only contributed towards resistance in lines derived from PI88788 and ‘Cloud’. At least 5.6 copies of the PI88788‐type *rhg1* were required to confer SCN resistance, regardless of the *Rhg4* (*GmSHMT08*) haplotype. However, when the *GmSNAP18* copies dropped below 5.6, a ‘Peking’‐type *GmSHMT08* haplotype was required to ensure SCN resistance. This points to a novel mechanism of epistasis between *GmSNAP18* and *GmSHMT08* involving minimum requirements for copy number. The presence of more *Rhg4* copies confers resistance to multiple SCN races. Moreover, transcript abundance of the *GmSHMT08* in root tissue correlates with more copies of the *Rhg4* locus, reinforcing SCN resistance. Finally, haplotype analysis of the *GmSHMT08* and *GmSNAP18* promoters inferred additional levels of the resistance mechanism. This is the first report revealing the genetic basis of broad‐based resistance to SCN and providing new insight into epistasis, haplotype‐compatibility, CNV, promoter variation and its impact on broad‐based disease resistance in plants.

## Introduction

Soybean cyst nematode (SCN, *Heterodera glycines* Ichinohe) is the most devastating pest among plant‐parasitic nematode species in the United States and worldwide. Annual soybean yield losses caused by this pest in the United States alone were estimated at $1.5 billion (Wrather and Koenning, 2006). The deployment of SCN resistance soybean varieties is the most efficient management practice to control the nematodes damage in soybean production areas. In past decades, many efforts have been made to evaluate the USDA Soybean Germplasm Collection for new sources of resistance to SCN. Over 100 plant introductions (PIs), including common accessions PI 88788, ‘Peking’ (PI 548402) and PI 437654 were identified as resistant to different SCN HG Types (Arelli *et al*., 1997, 2000; Concibido *et al*., 2004). Among these, PI 437654 and PI 567516C were highly resistant to multiple SCN races (Arelli *et al*., 2009; Brucker *et al*., 2005; Vuong *et al*., 2010; Wu *et al*., 2009).

To date, only two major sources of resistance lines, PI 88788 and ‘Peking’ have been commonly employed in soybean breeding programs (Bayless *et al*., 2018; Concibido *et al*., 2004). PI 88788 has eight copies at the *rhg1* locus and is the primary source used in commercial breeding programs to reduce SCN damage. More than 90% of SCN resistant cultivars are derived from this single source. A survey conducted in 2005 (Niblack *et al*., 2006) showed that 83% of the soybean fields in Illinois were infested with SCN and 70% of these had adapted to PI 88788, resulting in a reduction of the effectiveness when using SCN resistant cultivars as a crop management tool (Niblack *et al*., 2006). It is now urgent for soybean growers to have alternative sources of SCN resistance to overcome the selection pressure and the SCN population shifts.

Recent advances in high‐throughput genotyping and next‐generation sequencing technologies provide researchers with new opportunities to analyse genome structure at a large and a fine scale (Schmutz *et al*., 2014; Wang *et al*., 2014). Re‐sequencing of diverse genetic populations is a powerful approach for trait discovery and has been conducted in a variety of organisms including humans (Telenti *et al*., 2016), animals (Choi *et al*., 2015; Rubin *et al*., 2010; Zhou *et al*., 2016) and several other species (Aflitos *et al*., 2014; Lam *et al*., 2010, 2011; Varshney *et al*., 2017; Xu *et al*., 2012). Whole‐genome re‐sequencing (WGRS) facilitates the identification of functional variations and provides a comprehensive catalogue of genome‐wide polymorphism in closely related accessions. It also overcomes the limitation of missing data compared to other genotyping technologies (Jackson *et al*., 2011). Data from WGRS provides high resolution of the variation within populations, thus enabling marker‐assisted breeding, gene mapping and the identification of phenotype–genotype relationships. In humans, WGRS of diverse human populations aided the development of HapMap and facilitated the identification of common genetic variations (Gibbs *et al*., 2003). In crops such as rice (Huang *et al*., 2013; Yano *et al*., 2016), tomato (Aflitos *et al*., 2014), soybean (Lam *et al*., 2010), chickpea (Varshney *et al*., 2013), pigeonpea (Varshney *et al*., 2017) and maize (Gore *et al*., 2009), detailed analysis of re‐sequencing data provided a catalogue of genetic variants, such as single nucleotide polymorphisms (SNPs) and copy number variation (CNV), across the genome. This information has been used to identify genomic regions that are expected to play an important role during domestication and selection. CNVs are an important component of genetic variation influencing gene expression, phenotypic variation and adaptation by affecting genes and altering gene dosage (Redon *et al*., 2006; Sebat *et al*., 2004; Shlien and Malkin, 2009). In humans, CNVs are associated with cancer risk factors, neurological functions, regulation of cell growth and metabolism (Sebat *et al*., 2004).

In soybean, a large number of wild accessions, landraces and varieties have recently been re‐sequenced to provide useful information about genome structure and the discovery of new genes (Lam *et al*., 2010; Li *et al*., 2014; Qi *et al*., 2014; Schmutz *et al*., 2010; Valliyodan *et al*., 2016; Zhou *et al*., 2015). Moreover, the development of soybean high‐density markers from large sequencing data sets provides a powerful tool for whole‐genome prediction and selection applications (Patil *et al*., 2016). In SCN resistance, remarkable progress has been made since the cloning of the resistance genes that reside in the two major loci, *rhg1* and *Rhg4* (Cook *et al*., 2012; Lakhssassi *et al*., 2017a; Liu *et al*., 2012, 2017) . However, the mechanism of SCN broad‐based resistance and the interaction of these two loci in soybean accessions are still unclear and warrant further investigation. Therefore, we utilized the WGRS data from a diverse panel of 106 soybean accessions, including wild accessions, exotic germplasm, breeding lines and varieties, to investigate these two major SCN resistance loci using genome data mining approaches. These efforts provide new insight into the interconnectedness of haplotype compatibility, CNV, promoter variation and gene expression with broad‐based SCN resistance.

## Results

### Diversity, disequilibrium and signatures of selection at the *rhg1* and *Rhg4* loci

In soybean, two major SCN resistant QLTs have been identified on chromosomes 18 (*rhg1*) and 8 (*Rhg4*) (Cook *et al*., 2012; Liu *et al*., 2012; Vuong *et al*., 2010). To investigate the sequence diversity and disequilibrium of the *rhg1* and *Rhg4* loci, 1‐Mb regions on either side of these loci were analysed in 106 WGRS lines representing >96% of the sequence diversity (Valliyodan *et al*., 2016). The value of θπ, θ*w* and Tajima's D were estimated for related regions using sliding windows of 50‐kb and we observed extreme allele frequency differentiation over extended linked regions. As the location neared the *rhg1* locus, θπ increased greatly in the 100‐kb region (Figure S2a). The value of nucleotide diversity at the *rhg1* locus was approximately π = 0.00315, which was almost two times greater than the *G. max* average (0.00178) for all 106 lines. In contrast, a relatively low nucleotide diversity (θπ = 0.00159) at the *Rhg4* locus was observed (Figure S2a). Moreover, low nucleotide diversity was observed at both the *rhg1* and *Rhg4* loci if only *G. soja* (seven lines out of 106) was considered for analysis (Figure S2b), which could be attributed to the fact that SCN resistance was acquired during the domestication process of soybean. We also observed a higher *F*_st_ value (*P *< 0.005) associated with population differentiation near the *rhg1* locus when the multicopied *rhg1* genotypes were compared with single‐copy *rhg1* genotypes (Figure S2c). A relatively similar high *F*_st_ value (*P *< 0.01) was observed when the multicopied *Rhg4* genotypes were compared with single‐copy *Rhg4* genotypes. We further investigated linkage disequilibrium (LD) surrounding the *rhg1* and *Rhg4* loci. In agreement with Lee *et al*. (2015), the LD (measured by *r* ^*2*^) within the ˜200‐kb of the *rhg1* and *Rhg4* loci was strong and statistically significant, suggesting a block of strong LD extending to ~100‐kb on both sides of the *rhg1* and *Rhg4* loci (Figure S2d).

### Haplotypes grouping

**Figure 1:**
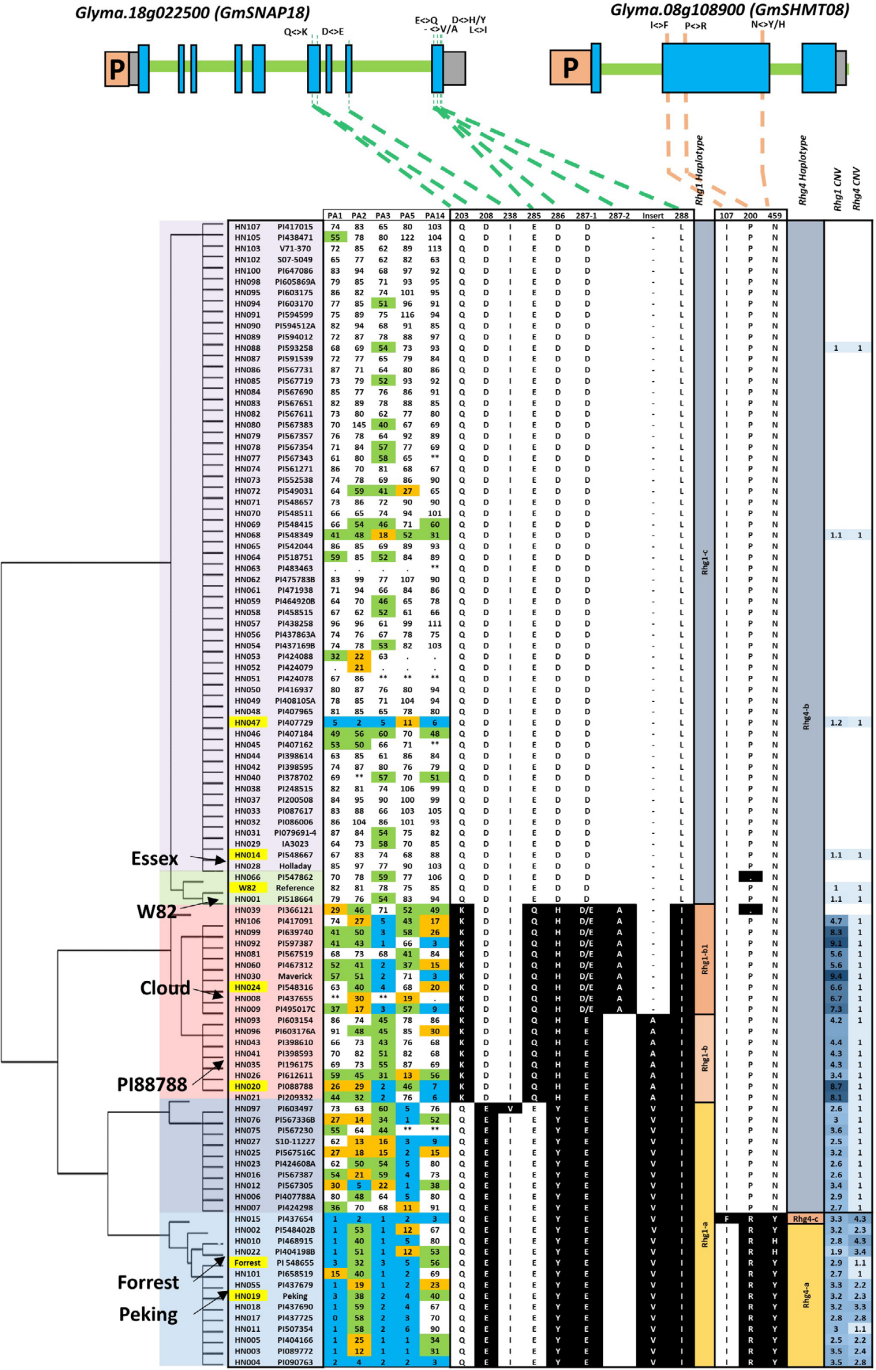
Haplotype clustering, correlation with female index and CNV of the *rhg1* and *Rhg4* locus in the 106 soybean lines. Schematic graphs show the position of amino acid change (non‐synonymous SNP/indel) for *Glyma.18g022500* (alpha soluble NSF attachment protein; a‐SNAP), and *Glyma.08g108900* (serine hydroxymethyl transferase; SHMT) genes. The SNPs in black background are different to the reference genome (‘Williams 82’). In the gene model diagram (top of the figure), the blue box represents exons, green bar represents introns, orange box represents promoter region and grey box represents 3′ or 5′ UTR. SNPs were positioned relative to the genomic position in the genome version W82.a2. SCN Female index ratings are shown for each genotype × race combination (races include PA1, PA2, PA3, PA5 and PA14). Female index ratings are shared according to the degree of resistance/susceptibility; scores of 0–9 were resistant (blue shading); 10–29 were moderate resistant (orange shading); 30–59 were moderate susceptible (green shading) and >60 were susceptible (no shading). [Colour figure can be viewed at wileyonlinelibrary.com]

The genetic diversity at SCN resistance loci provided an opportunity to obtain an overview of the haplotype variation at both the *rhg1* and *Rhg4* loci. As reported earlier, three genes (*Glyma.18g022400, Glyma.18g022500 and Glyma.18g022600*) at the *rhg1* locus together confer resistance to SCN in PI 88788 (Cook *et al*., 2012). Despite a high number of sequence polymorphisms found within each *rhg1* repeat in SCN‐resistant lines, the SNPs that cause an altered amino acid sequence (non‐synonymous) were identified only in the *Glyma.18g022500* (*GmSNAP18*) gene (Figure 1). Three major haplotypes; *rhg1‐a*,* rhg1‐b* and *rhg1‐c* were identified for the *GmSNAP18* gene based on nine amino acid sequences changes (Q203K, D208E, I238V, E285Q, D286Y/H, D287(1)E, ‐287(2)A, ‐287(3)V/A and L288I) (Figure 1). The *rhg1‐c* corresponds to ‘Williams 82’‐like *rhg1*. The second haplotype was divided into *rhg1‐b* (similar to PI 88788‐type lines) and *rhg1‐b1* (similar to ‘Cloud’ type lines). Based on read depth across the known repeat and flanking regions, 45 lines were examined for CNV and showed an estimated *rhg1* copy number greater than one. The average number of copies across all tested lines was 3.6, with the highest at 9.4 for Maverick (Figure 1 and Figure S3A). Moreover, a wide range of DNA variation was observed at the *rhg1* locus, including SNPs, insertion and deletion polymorphisms. Across the 25.1‐kb interval, there was an average of 130 polymorphisms per accession compared with the soybean reference genome (Table S1). The patterns of amino acid variation at each *rhg1* genotype were highly correlated with the copy number and response to different SCN races. For example, the three major haplotype groups include high‐copy *rhg1* (PI 88788‐type, copy number from 2.9 to 9.4), low‐copy *rhg1* (‘Peking’‐type, copy number from 1.9 to 3.5) and single‐copy *rhg1* (Table 1, Figure 1). The lines with high CNV exclusively carry the PI 88788‐type of SNP variants and the lines with low CNV exclusively carry ‘Peking’‐type of SNP variants. The lines with single copy *rhg1* do not carry any PI 88788‐ or ‘Peking’‐type of SNPs and are known to be susceptible to SCN.

**Table 1:**
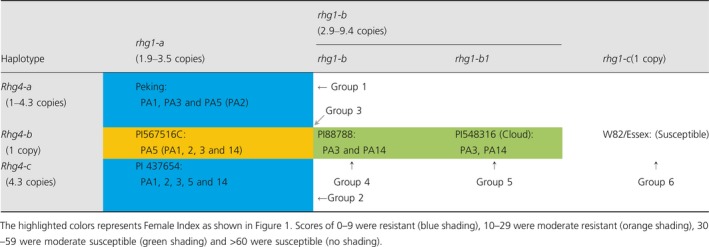
Genetic basis of haplotype to haplotype interaction of *rhg1* and *Rhg4*

Similar to the *rhg1* locus, we performed analysis of the sequence variation, CNV and haplotypes at the *Rhg4* locus encompassing three genes (*Glyma.08g108800, Glyma.08g108900 and Glyma.08g109000*). The gene *Glyma.08g108900,* encoding Serine hydroxymethyltransferase (*GmSHMT08*), showed three non‐synonymous SNPs associated with the SCN reaction (Figure 1). In the earlier soybean reference genome assembly W82.a1, *GmSHMT08* (alias *Glyma08g11490*) was predicted to produce 503 amino acids, whereas in the most current assembly W82.a2 (Song *et al*., 2016) the primary transcript was 573 amino acids long. The first 70 amino acids in the assembly W82.a1 were missing, and this could be caused by an alternative splicing event or exon skipping. The CNV analysis showed the presence of multiple copies (1–4.3) of *Rhg4,* which were strongly associated with the non‐synonymous SNPs leading to P<>R and N<>Y/H (Figure 1). The highest number of *Rhg4* copies was observed in PI 468915 and PI 437654. The average number of *Rhg4* variant sites per soybean line was estimated to be 51 for multicopy *Rhg4* lines, and 26 for the single‐copy *Rhg4* lines in a 21.3‐kb interval compared to the reference genome (Table S2). Based on amino acid variants, the *Rhg4* locus was broadly divided into two haplotypes, the *Rhg4‐b* (W82‐like *Rhg4*) and *Rhg4‐a* (‘Peking’‐type *Rhg4*). PI 437654 carried additional non‐synonymous SNPs leading to an I<>F amino acid change which we named haplotype *Rhg4‐c* (Figure 1).

To further confirm the estimated CNV using WGRS data of both *rhg1* and *Rhg4* loci (Figure S4), we performed additional experiments, including digital polymerase chain reaction (PCR), Taqman assays and microarray‐based comparative genomic hybridization (CGH) analysis (Table 2; Figure S5). Seven lines with known SCN resistance were selected for the verification of copy number at both *rhg1* and *Rhg4* loci. The reported CNV data (Cook *et al*., 2012) for ‘Peking’, PI 88788, ‘Forrest’, PI 438489B and PI 437654 were taken into consideration for comparison. Highly consistent results were observed across different platforms as well as earlier published studies (Table 2). Results obtained from our study provide the first report showing the presence of CNV at the *Rhg4* locus, directly impacting SCN resistance. Having established that both *rhg1* and *Rhg4* have complex genomic and functional structures, we sought to better resolve how the structural and functional properties interact in determining SCN resistance of soybean.

**Table 2:** A comparison and confirmation of the *rhg1* and *Rhg4* CNV using different platforms from representative SCN‐resistant lines

Genotype	Seq id	*rhg1*				*Rhg4*			
		WGRS	Digital PCR	Taqman assay	CGH	WGRS	Digital PCR	Taqman assay	CGH
Peking	HN019	3.15	2.72	3.14	2.72	2.35	1.48	1.54	1.79
S05‐11482	HN013	3.16	2.98	3.01	2.68	1.06	1.06	0.98	0.95
PI088788	HN020	8.73	8.53	8.22	7.60	1.01	1.02	0.95	1.00
PI438489B	–	3.33	3.35	3.28	2.68	3.12	2.56	3.22	1.85
PI437654	HN015	3.30	3.6	3.37	2.71	4.34	4.7	3.49	3.47
Magellan	–	1	1	0.97	0.97	1.04	1	0.93	0.95
Forrest	–	2.89	–	–	2.66	1.02	–	–	0.94

CGH, comparative genome hybridization; WGRS, whole‐genome re‐sequencing.

### SCN epistatic interaction between *rhg1* and *Rhg4* loci

Haplotype analysis revealed that only three non‐synonymous SNPs at the *GmSHMT08* gene had a strong association with both CNV of *Rhg4* loci and SCN resistance (Figure 1). In this study, mutational analysis was employed to study the impact of the three reported haplotypes representing the 106 sequenced soybean lines for important catalytic, substrate binding, structural stability and subunit interaction sites within the GmSHMT08. The structure‐based homology modelling was conducted with the ‘Forrest’ genotype, which carries three amino acid changes and also lacks the first 70 amino acids, suggesting that the first 70 amino acids do not affect the *GmSHMT08* gene's function in resistance to SCN. The presence of 70 amino acids could be due to alternate splicing or exon skipping and these 70 amino acids might also have a role in organelle targeting, which warrants further study. The structure‐based homology modelling analysis provided an interesting platform to study the differences between the resistant and susceptible haplotypes at GmSHMT08 (Karthikraja *et al*., 2009). Thus, the possible impact of each mutation on the predicted GmSNAP18 and GmSHMT08 structures were analysed.

**Figure 2:**
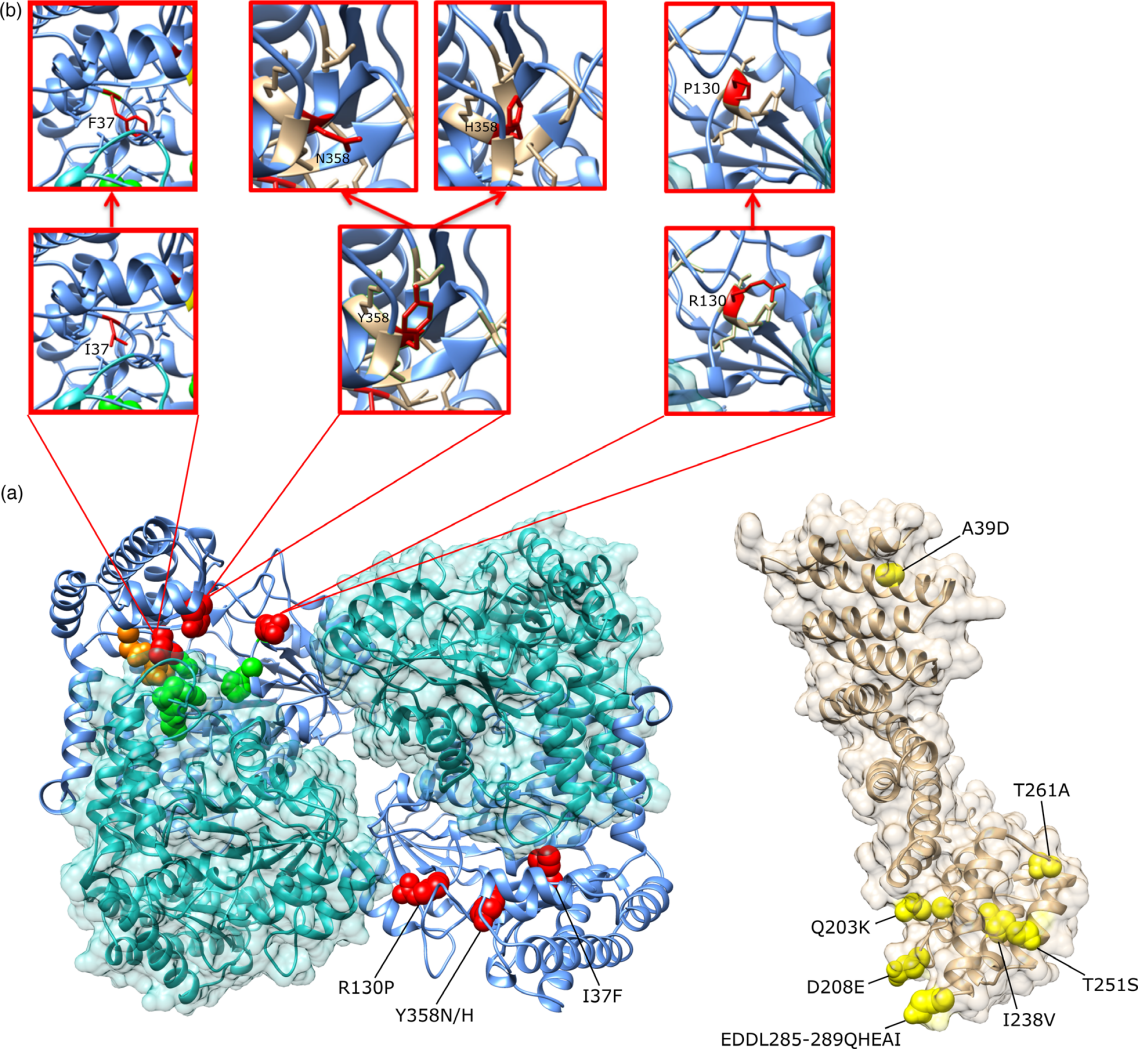
Homology modelling of the GmSNAP18 and the tetrameric GmSHMT08 from ‘Forrest’ (‘Peking’‐type resistance). (a) GmSHMT08 tetramer showing the characterized three spontaneous mutation sites, I37F, R130P and Y358N/H (red) as well as GmSNAP18 showing the seven characterized haplotypes (yellow) between resistant and susceptible soybean lines from the 106 accessions. Selected cofactor binding residues, S39, Y59, G132, H134 and R250 (Green) and selected dimerization residues E35 and E40 (Orange) are also displayed. (b) The three GmSHMT08 mutations induced on the prediction model to explore their effects. [Colour figure can be viewed at wileyonlinelibrary.com]

The R130P polymorphism (corresponding to P200R in Figure 1) is localized close to the pyridoxal phosphate (PLP) cofactor binding. This site was specific to *Rhg4‐a* and *Rhg4‐c* alleles in SCN resistant lines and occurs in 15.1% of the sequenced soybean lines. The amino acid change R130P (P200R in Figure 1) from a positively charged arginine residue to an uncharged proline residue may impact this cofactor binding (Figure 2). The second GmSHMT08 polymorphism (Y358N; N459Y in Figure 1) represents 13.4% of the sequenced soybean resistant lines. This polymorphism resides within a pocket near the catalytic and substrate binding site of the GmSHMT08 protein, with a mutation directly altering the negatively charged hydrophobic tyrosine residue into a polar uncharged asparagine residue. This change may cause a stearic conflict with other residues shown within the 5 Å region around the Y358N polymorphism (Figure 2). At the same site, a small fraction of the sequenced resistant soybean lines (1.98%) carried the Y358H (N459H in Figure 1) natural mutation. Lastly, one soybean line among the 106 sequenced lines, PI 437654, carried a polymorphism at I37F (I107F in Figure 1). This amino acid change between two different sized hydrophobic side chains; phenylalanine and isoleucine, may also present conflicts with the other residues as shown within the 5 Å analysed area (Figure 2).

### Identification of tandem repeats at the *Rhg4* locus

Based on the WGRS information, the genomic region surrounding the cloned *Rhg4* gene *GmSHMT08* (Liu *et al*., 2012) appeared to be duplicated in at least 11 of the 106 sequenced genomes (Figure 1). This finding was confirmed in ‘Peking’, PI 437654 and PI 438489B using a combination of CGH, DPCR and Taqman assays (Table 2). The duplicated region was estimated to be approximately 30‐kb (Figure S6). To confirm whether the duplications were present in these lines and to reveal their sizes and locations, we designed three sets of primers based on the reference genome of ‘Williams 82’ to see whether we could amplify 16.7‐kb, 20.6‐kb and 24.8‐kb regions flanking the cloned *Rhg4* gene. Results obtained hypothesize that if two primers are located inside a complete duplicated region, a PCR product of the expected size defined by the primers should be generated. After the PCR amplification, a PCR band of the expected size was detected in ‘Williams 82’, ‘Peking’ and PI 437654 for all three primer sets respectively (Table S3). These results suggest that these primers as well as the regions defined by them are located inside a duplicated region (if such a duplication exists in a given genotype), and that the duplicated region or repeat should be longer than the 24.8‐kb region.

**Figure 3:**
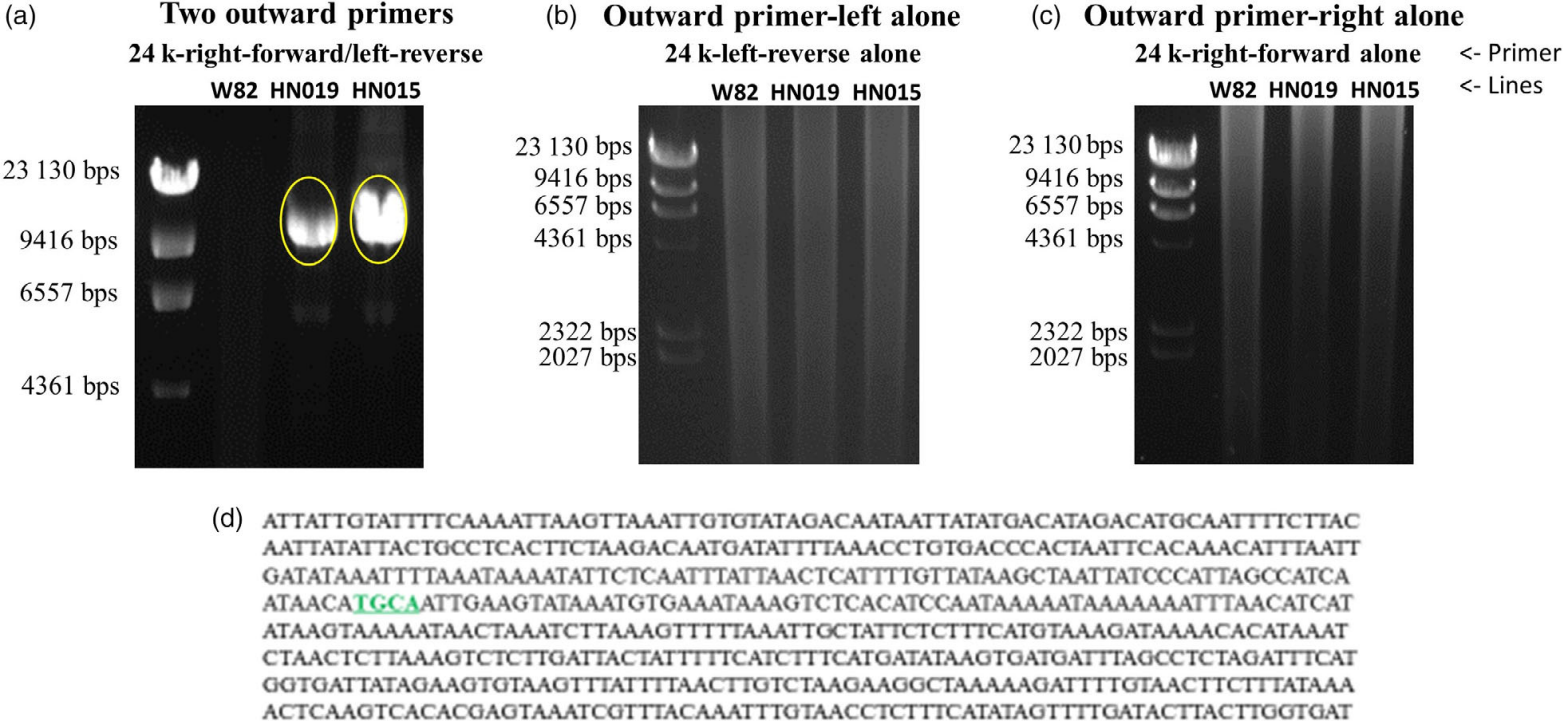
Amplification of the junction regions between two neighbouring repeats in ‘Williams 82’, ‘Peking’ (HN019) and PI 437654 (HN015) soybean lines. (a) Gel image of the PCR bands obtained for the junction between two neighbouring tandem repeats. (b) Gel image of the PCR reactions intended to amplify the regions between two neighbouring back‐to‐back inverted repeats if present. (c) Gel image of the PCR reactions intended to amplify the regions between two neighbouring head‐to‐head inverted repeats. (d) Part of the sequence obtained from sequencing the PCR products circled in yellow in (a), showing the joining of two sequences from two different regions in the sequenced ‘Williams 82’ reference genome, separated by the extra four bps, TGCA (underlined and highlighted in green). The sequences from both ‘Peking’ and PI 437654 were the same. [Colour figure can be viewed at wileyonlinelibrary.com]

Since this 24.8‐kb length is rather close to the estimated 30‐kb duplicated region, we speculated that the ends of this 24.8‐kb region were close to the junction between two neighbouring repeats. If this is the case, we would be able to amplify this junction region by PCR in line with duplications using two outward end primers of the 24.8‐kb region as depicted graphically in Figures S6 and S7. However, these primers should fail to amplify in ‘Williams 82’, which does not have any duplication at the *Rhg4* locus. Indeed, a PCR band of approximately 11‐kb was generated in both ‘Peking’ and PI 437654, but not in ‘Williams 82’, when both primers were included in the reactions (Figure 3). No PCR bands were generated in any lines when a single outward primer was used in the reactions, which were intended to amplify the junctions between two neighbouring inverted (either back‐to‐back or head‐to‐head) repeats (Figure 3). After sequencing the purified PCR products from both lines, two sequences from different locations of the reference genome were linked with each other and separated by the following four base pairs, TGCA (Figure 3). The joining of two sequences from different regions in these lines indicated that duplications or sequence arrangements were present. To confirm that the junction sequence was not due to PCR artefacts, two primers were designed to flank an 819‐bp junction region and were used in PCR reactions on genomic DNA from different soybean lines. After PCR amplification, a PCR band of approximately 800 bp was detected in ‘Peking’, PI 437654 and PI 438489B, but not in ‘Williams 82’. The sequences obtained from these PCR products matched the initially identified junction sequence (Figure S8). Therefore, we concluded that repeats were present in these lines and the TGCA sequence upstream should correspond to the end of one repeat and the TGCA sequence downstream should be the beginning of the neighbouring tandem repeat (in the same orientation as 24.8‐kb region). By aligning the beginning and end sequences with the reference genome, we found that the repeat at the *Rhg4* locus in ‘Peking’, PI 437654 and PI 438489B was 35,705 bp (Figure S9). According to the reference genome, this repeat contains the following four genes, *Glyma.08g108800* (Adenosylhomocysteinase), *Glyma.08g108900* (the cloned *Rhg4*, encoding a serine hydroxymethyltransferase, SHMT), *Glyma.08g109000* (encoding a proprotein convertase subtilisin/kexin) and *Glyma.08g109100* (encoding a NAD‐dependent epimerase/dehydratase) (Figure S9). It should be noted that the PCR analysis provides the structural map for at least one junction in the tandem repeat arrangement, but does not confirm that all copies from all of the genotypes have the same structure.

### *Rhg4* copy number and broad‐based resistance to SCN

The presence of CNV for the *rhg1* locus is common (or frequent) when compared to the *Rhg4* locus (Figure 1; Table S4) and the PI 88788 source carrying high copies of *rhg1* is in over 95% of existing SCN resistant varieties marketed in the United States. However, the PI 88788‐type resistance has been broken due to adaptation in SCN populations to reproduce on lines derived from PI 88788. Several lines carrying the haplotypes *rhg1‐b* or *rhg1‐b1*, and having greater than 5.6 copies of the *GmSNAP18* showed SCN resistance to race 3 and 14. The remaining line with *rhg1‐b* or *rhg1‐b1* but less than 5.6 *rhg1* copies were susceptible to 3–4 SCN races, except PI 417091 (Figure 1). Thus, a copy number of 5.6 of *rhg1* can be hypothesized to be the threshold for resistance to both races 3 and 14. These lines do not carry CNV or a non‐synonymous mutation in the *GmSHMT08* gene. However, lines carrying ‘Peking’‐type *rhg1* (*rhg1‐a* haplotype) with relatively lower copies (1.9–3.5) showed resistance to multiple SCN races. This is because these lines also carry CNV and/or retained non‐synonymous mutations in *GmSHMT08* (i.e. *Rhg4‐c* and *Rhg4‐a*) (Figure 1; Table 1). For example, PI 567516C carries the *rhg1‐a* allele, but also carries the wild‐type allele at *Rhg4* (*Rhg4‐b*), and hence showed moderate resistance to multiple races. However, a line (e.g. PI 437654) carrying multiple copies of *Rhg4* in addition to *rhg1‐a* oftentimes showed resistance to all five races. From these observations, we concluded that in addition to the ‘Peking’‐type *GmSNAP18* with 2–4 copies, the CNV and non‐synonymous SNPs in the *GmSHMT08* gene play a paramount role for resistance to multiple races.

Based on epistatic interactions of the *GmSNAP18* and *GmSHMT08*, the 106 soybean lines were grouped into six categories that showed strong associations between genotypic variation (CNV and non‐synonymous changes) and nematode susceptibility/resistance phenotypes (Table 1 and Table S4). The lines of group‐1 and ‐2 (*rhg1‐a *+ *Rhg4‐a* and *rhg1‐a *+ *Rhg4‐c*, respectively) carry only ‘Peking’‐type of *rhg1* and *Rhg4* and were highly resistant to race 1, 2, 3, 5 and resistant or moderate resistant to race 14. Lines belonging to group‐3 (*rhg1‐a *+ *Rhg4‐b*) carry only ‘Peking’‐type *Rhg1* and conferred resistance to race 5. The group 4 and 5 (*rhg1‐b *+ *Rhg4‐b* and *rhg1‐b1 *+ *Rhg4‐b*, respectively) lines carry only PI 88788/‘Cloud’‐type of the *rhg1* and showed greater resistance to races 3 and 14. A comparison of PI 88788 and ‘Cloud’‐type *rhg1* indicated that the lines with the ‘Cloud’‐type of *rhg1* had better resistance to SCN. The lines belonging to the group‐6 (*Rhg1‐c *+ *Rhg4‐b*) carry ‘Williams 82’‐type loci and hence were highly susceptible to all five SCN races (Table S4). Surprisingly, PI 407729 (a group 6 line) does not carry the above‐mentioned resistant loci (non‐synonymous SNP and CNV), but exhibited moderate to high resistance to all five races. These observations suggest that this line may contain novel resistance loci that confer SCN resistance independent of *rhg1* and *Rhg4*. To infer the resistance mechanism in PI 407729, we analysed *GmSHMT08* and *GmSNAP18* promoter haplotypes as discussed in the next sections.

### Variation in *GmSHMT08* and *GmSNAP18* promoters in combination with CNV confers additional level of resistance to SCN

**Figure 4:**
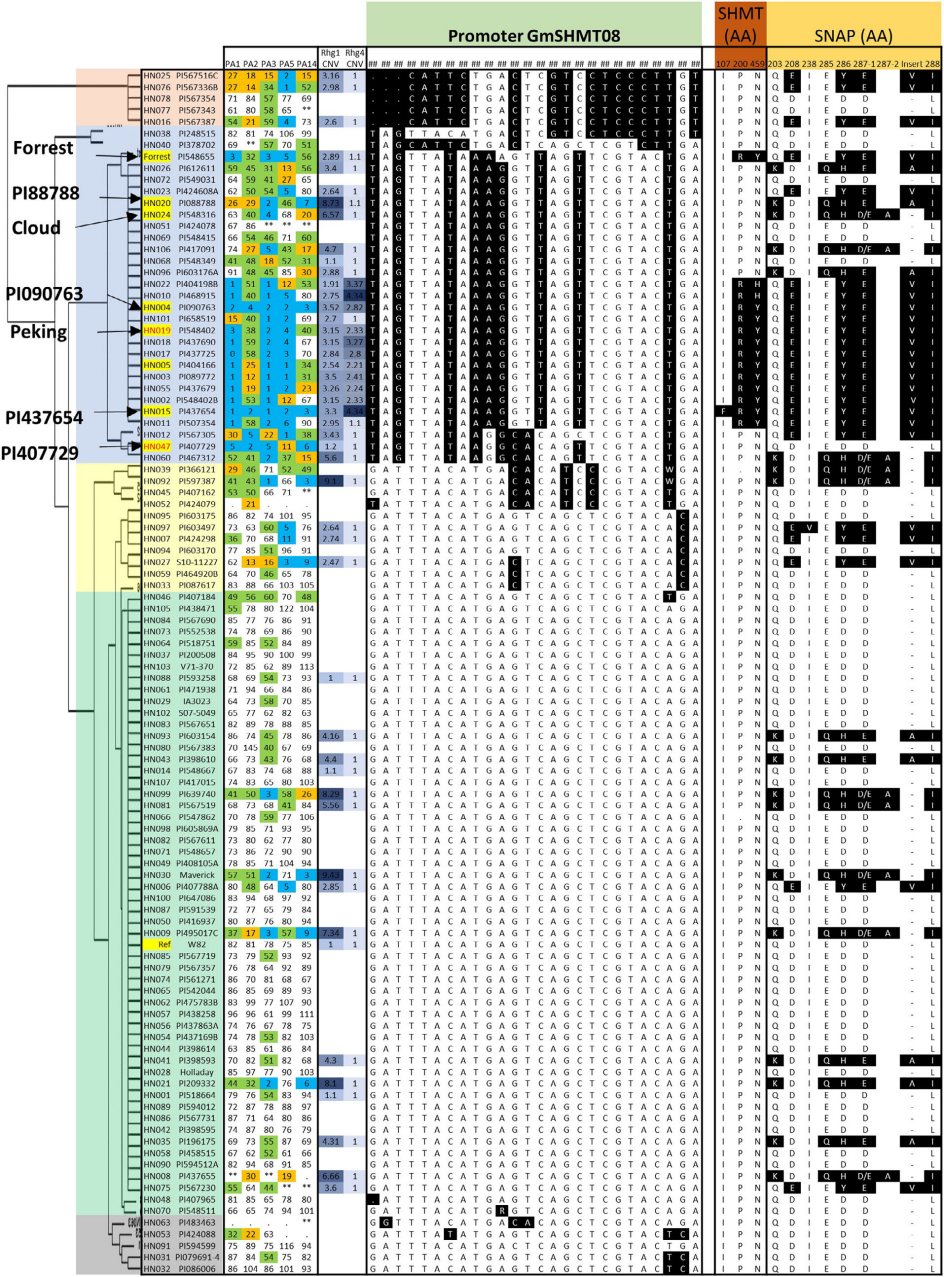
Haplotype clustering of *GmSHMT08* promoter. Schematic graph shows correlation with female index and amino acid changes of the *GmSHMT08* and *GmSHAP18 protein* in 106 soybean lines. SNP in black background are different to the reference genome (‘Williams 82’). SNPs were positioned relative to the genomic position in W82.a2. SCN Female index rating system: FI = 0–9, resistant (blue shading); 10–29 moderate resistance (orange shading); 30–59 moderate susceptibility (green shading); >60, susceptible (no shading). [Colour figure can be viewed at wileyonlinelibrary.com]

**Figure 5:**
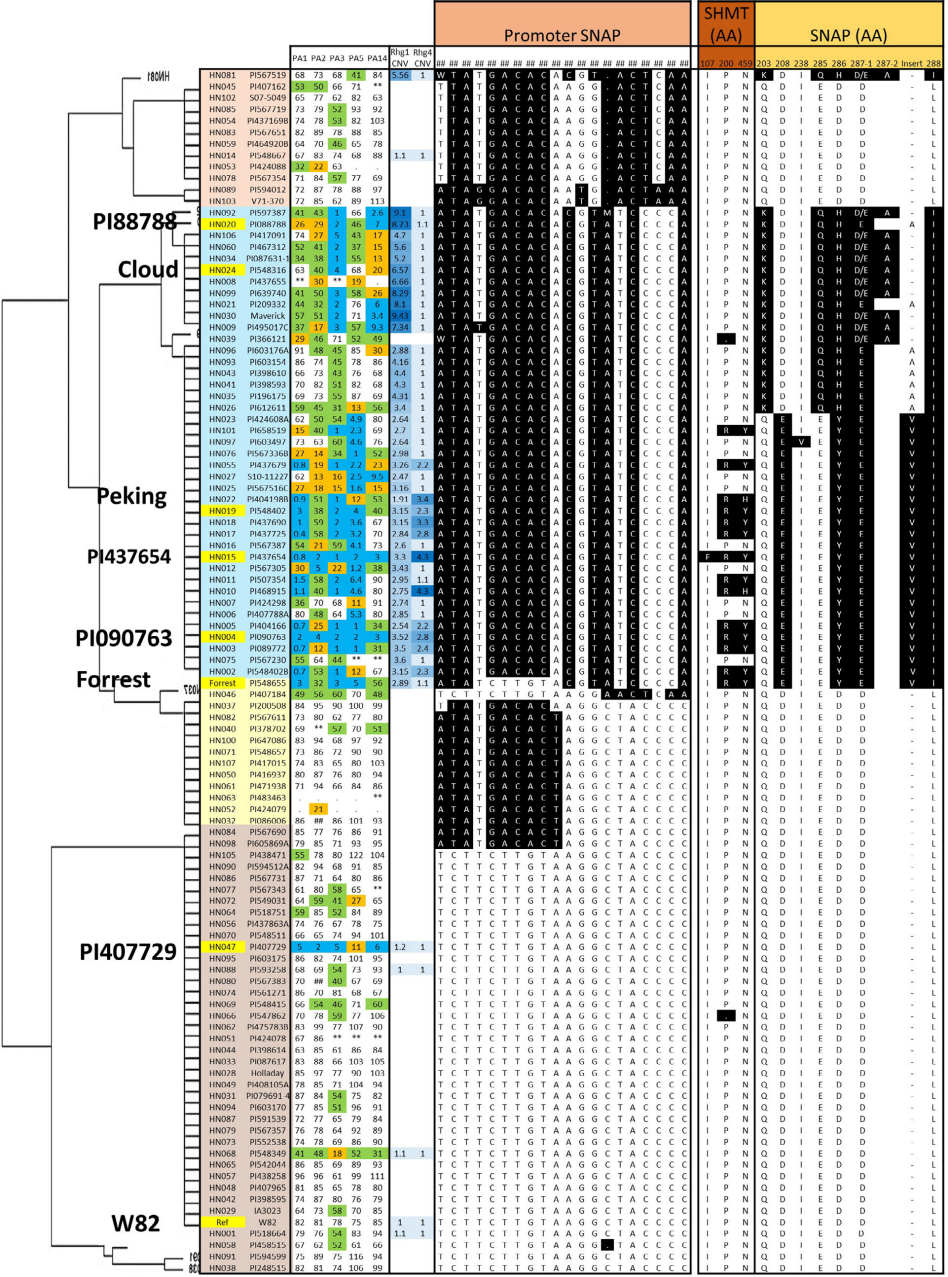
Haplotype clustering of *GmSNAP18* promoter. Schematic graph shows correlation with female index and amino acid changes of the *GmSHMT08* and *GmSHAP18 protein* in 106 soybean lines. SNP in black background are different to the reference genome (‘Williams 82’). SNPs were positioned relative to the genomic position in W82.a2. SCN Female index rating system: FI = 0–9, resistant (blue shading); 10–29 moderate resistance (orange shading); 30–59 moderate susceptibility (green shading); >60, susceptible (no shading). [Colour figure can be viewed at wileyonlinelibrary.com]

It is well documented that SNPs in the promoter region, including the 5′ UTR, can abolish gene function, expression level and localization (Patil *et al*., 2015). Recently, Bayless *et al*. (2018) reported ~300‐bp deletion within the promoter of another *SNAP* paralog gene (*Glyma.11G234500*; α‐SNAP Ch11‐ IR) in ‘Forrest’ genotype. The authors cloned the WT locus of the *GmSNAP11* from W82 with native promoter and terminator and observed elevated SNAP protein expression in transgenic roots. In the current study, we have shown that resistant alleles contain nine and three natural point mutations in the GmSNAP18 and GmSHMT08 proteins, respectively, when compared to the susceptible alleles. Out of the 106 lines examined, 14 lines carry resistant alleles at both the *rhg1‐a* and the *Rhg4‐a/Rhg4‐c* haplotypes, corresponding to the ‘Peking’‐type of resistance. However, the other 30 SCN resistant lines, corresponding to both ‘Cloud’‐ and PI 88788‐type of resistance, carry the resistant *rhg1‐a* (11 lines), *rhg1‐b* (eight lines) and *rhg1‐b1* (11 lines) haplotype, but all contain the *Rhg4‐b* susceptible allele. Interestingly, PI 407729 carries both susceptible alleles at the *rhg1‐c* and the *Rhg4‐b* loci, but exhibited resistance to all five races. In order to gain more insight into SCN resistance in this line, we performed a haplotype analysis clustering of all the 106 lines at the promoter level of both genes (Figures 4 and 5).

**Figure 6:**
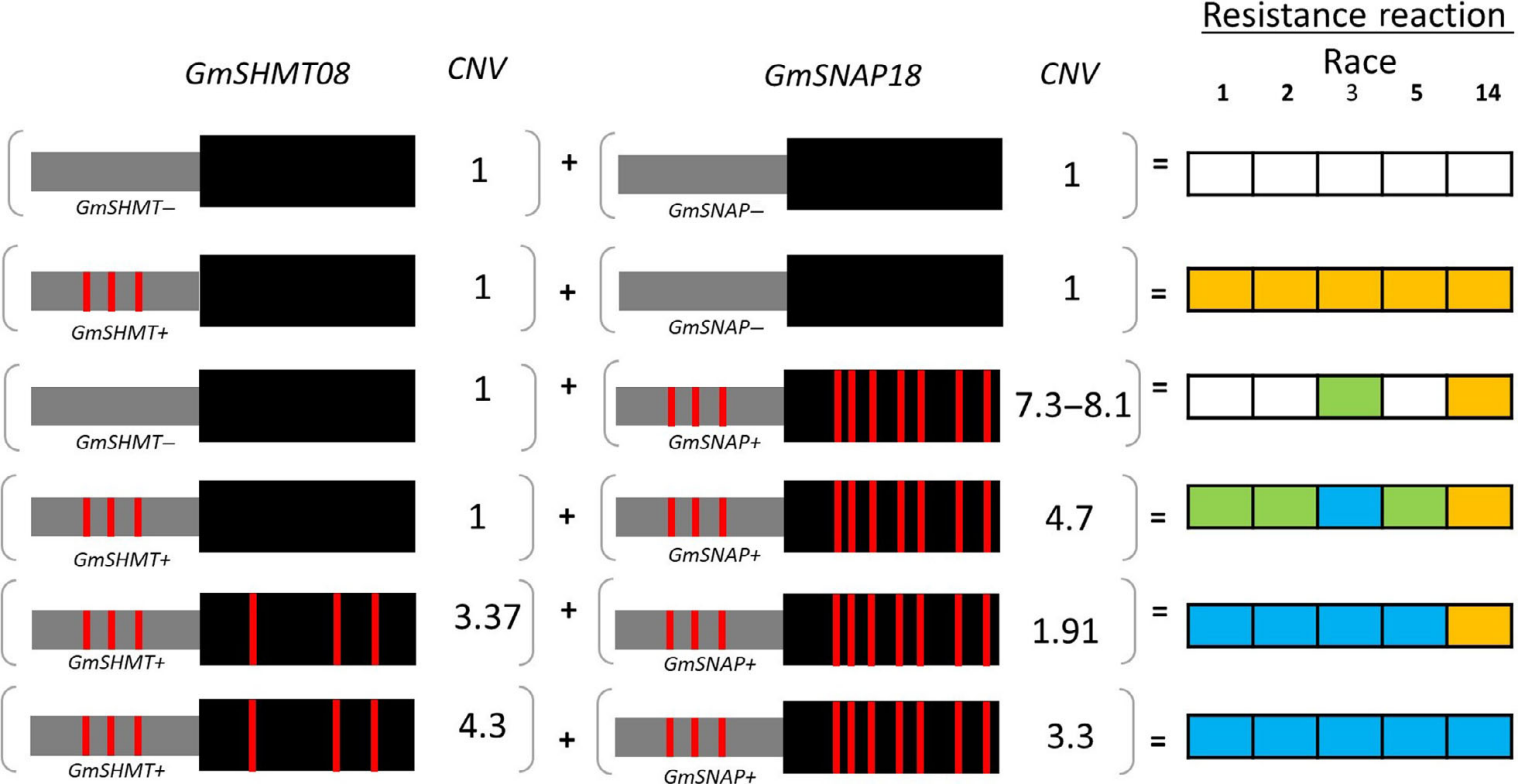
Schematic overview of allelic variants (promoter, amino acid change, CNV) in *GmSHMT08* and *GmSNAP18* genes and their impact of SCN resistance in five races. SCN Female index rating system: FI = 0–9, resistant (blue shading); 10–29 moderate resistance (orange shading); 30–59 moderate susceptibility (green shading); >60, susceptible (no shading). Grey box represents promoter region; black box represents coding region and vertical red lines represents amino acid change. (Not drawn to the scale). [Colour figure can be viewed at wileyonlinelibrary.com]

The analysis suggested an additional layer for the resistance mechanism. The haplotype of the *GmSHMT08* promoter region (~3.8‐kb) showed that most of the resistant lines carry a unique haplotype, which was different from that of the SCN susceptible lines. The analysis substantiated that PI 407729 carries several SNPs and Indels in the promoter region that are different from the susceptible lines ‘Williams 82’ and ‘Essex’, but similar to the promoters of the resistant lines (*GmSHMT08* ^*+*^) ‘Forrest’, ‘Peking’, PI 88788 and PI 437654. This observation suggests that the SNPs/indels identified in the *GmSHMT08* ^+^ promoter may be responsible for SCN resistance in PI 407729 (Figures 4 and 6). Notably, copy numbers of 3.4 and 4.7 were enough to confer broad‐based resistance to SCN when the *GmSHMT08* ^+^ promoter was present. However, if a given soybean line lacked the *GmSHMT08* ^*+*^ promoter, then at least 8.1 and 7.3 copies of the *GmSNAP18* (*rhg1*) were required to confer resistance in PI 88788‐ and ‘Cloud’‐type‐*rhg1* respectively (Table S5). Similarly, in ‘Peking’‐type lines, 1.91 copies of *rhg1* were enough to confer SCN resistance when the *GmSHMT08* ^+^ promoter was present. However, when the promoter variation (*GmSHMT08* ^‐^) was present, the *rhg1* copy number should be at least 2.47 to confer resistance to SCN (Figures 4, 5, 6; Table S5).

The haplotype analysis of the *GmSNAP18* promoter (~1.5‐kb) showed that the majority of the resistant lines carry a specific promoter haplotype (Figures 5 and 6). In addition, lines that lacked this promoter haplotype were susceptible to SCN. Four lines PI 196175, PI 398593, PI 398610 and PI 603154 carried both the resistant loci (non‐synonymous SNP and CNV at the *rhg1* locus) and promoter haplotype but were susceptible to SCN. This can be explained by the presence of the susceptible *GmSHMT08* ^‐^ promoter. Overall, these results suggest that variants (SNP/indel) within the promoter region coupled with CNV provides an additional layer of resistance, and the susceptible lines may be converted into resistant by replacing the susceptible promoter with the *GmSHMT08* ^+^ version (Figure 6).

### Expression analysis and *Rhg4/rhg1* copy number variants

**Figure 7:**
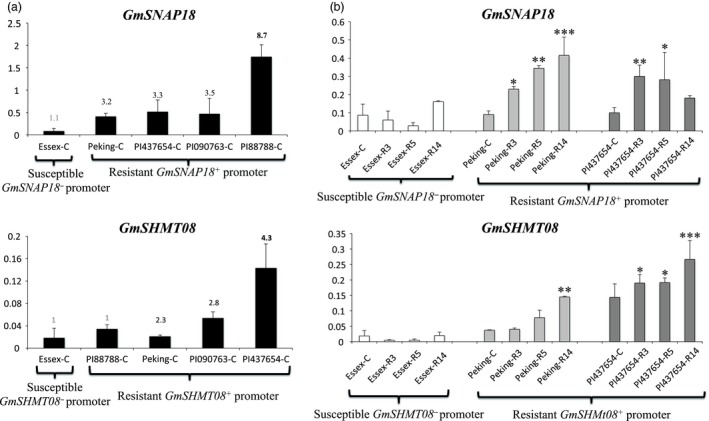
Quantitative RT‐PCR analyses of *GmSNAP18* and *GmSHMT08* in the roots at 2 days in the absence (a) and the presence (b) of SCN infection. (a) Roots at 2 days without SCN infection were used as control. (b) Three SCN races were used (PA3, PA5 and PA14). Five indicator lines representing the CNV and haplotype combinations at the promoter and amino acid sequence of the predicted GmSNAP18 and GmSHMT08 were selected. These lines include ‘Peking’, PI 437654, PI 090763 and PI 88788 lines that carry the resistant *GmSHMT08* and *GmSNAP18* promoters (all these four lines deemed resistant to SCN). However, ‘Essex’ carries the susceptible *GmSHMT08* and *GmSNAP18* promoter and is susceptible to SCN. Three biological replicates were performed for each line. Numbers on the top of each graph represent the line copy number. The error bar stands for the s.e.m. Asterisks indicate significant differences between samples as determined by ANOVA (****P < 0.0001 and **P < 0.01).

To gain more insight into the impact of the identified CNV on both the *GmSNAP18* and *GmSHMT08* transcripts, qRT‐PCR analysis was carried out in a number of lines representing different subgroups. Based on the haplotype combinations and CNV, five indicator lines including ‘Essex’, ‘Peking’, PI 437654, PI 90763 and PI 88788 were selected, and screened in the presence and in the absence of the nematode infection (Table S6). In the absence of SCN infection, expression analysis shows that the *GmSNAP18* root transcripts in five indicator lines correlated perfectly with their *rhg1* CNV (Figure 7a). In fact, *GmSNAP18* transcripts in PI 88788, which has the highest copy number (8.7) of *rhg1,* were 2.70, 2.34, 3.24 and 20.75 times more abundant when compared to PI 90763 (copy number = 3.5), PI 437654 (copy number = 3.3), ‘Peking’ (copy number = 3.2), and ‘Essex’ (copy number = 1.1), respectively. Overall, *GmSNAP18* transcripts were up to 10‐fold more abundant than the *GmSHMT08* transcripts. Notably, the tested lines also carried SNP in the *GmSHMT08* ^*+*^ promoter (Figure 7a). In the case of *GmSHMT08*, PI 437654 has the highest *Rhg4* copy number (4.3) and exhibited 1.8 and sixfold more abundant transcripts when compared to PI 90763 (copy number = 2.8), and ‘Peking’ (copy number = 2.3), respectively. In addition, PI 437654 transcripts were 13‐fold more abundant than ‘Essex’ (copy number = 1) carrying the susceptible *GmSHMT08* ^*‐*^ promoter. In summary, results show that both variations in promoter sequence and gene copy number are associated with the differences in *Rhg4* gene expression.

Recently, it was shown that *GmSNAP18* transcripts were induced in ‘Forrest’ (carrying the *rhg1‐a* and *Rhg4‐a* haplotypes) and PI 88788 (carrying the *rhg1‐b* and *Rhg4‐b* haplotypes) in response to SCN infection, whereas the susceptible line ‘Essex’ (carrying the *rhg1‐c* and *Rhg4‐b* haplotypes) exhibited very low mRNA levels of *GmSNAP18* (Liu *et al*., 2017). In ‘Forrest’, *GmSNAP18* transcripts showed about a twofold up‐regulation in SCN‐infected roots compared to noninfected roots at 3 and 5 days post‐SCN infection (dpi). Similarly, in PI 88788 *GmSNAP18* transcripts showed a twofold up‐regulation in SCN infected roots compared to the noninfected control at 5 dpi. *GmSHMT08* transcripts were also found to be induced in both ‘Forrest’ and PI 88788 soybean lines (Kandoth *et al*., 2017). Similarly, we investigated the expression of ‘Essex’, ‘Peking’ and PI 436754 in response to infection by three SCN races (PA3, PA5 and PA14) at 2 dpi. The analysis demonstrated that *GmSNAP18* transcripts (underlying *rhg1‐a* haplotype) were induced in the presence of the three nematode races in both ‘Peking’ and PI 436754 (Figure 7b). In summary, all the resistant lines harbouring *rhg1‐a, rhg1‐b*,* Rhg4‐a*,* Rhg4‐b* and *Rhg4‐c* haplotypes, exhibited abundant transcripts in the absence of SCN infection, a finding that correlates with the CNV in these lines. In addition, their transcript levels were further induced in the presence of the three SCN races tested. However, susceptible lines like ‘Essex’ with reduced copy number (*rhg1‐c* = 1.1 and *Rhg4‐b* = 1) exhibited the lowest expression level and absence of any induction of the *rhg1‐c* nor *Rhg4‐b* transcripts.

## Discussion

The current study utilized high‐quality deep sequencing information (~15× genome coverage) for the *rhg1* and *Rhg4* loci and identified haplotypes associated with SCN resistance to five races. Our haplotype analysis also identified the presence of SNPs associated with CNV. We obtained nearly identical results for CNV of the *rhg1* locus*,* which is also related to the SCN‐resistance efficacy, as previously reported (Cook *et al*., 2012). Unprecedentedly, we observed increased copy number of the *Rhg4* gene in 11 soybean lines, ranging from 1.2 to 4.3 copies. The copy number increases were confirmed using different molecular platforms, including digital‐PCR, Taqman assay and CGH. Furthermore, we also confirmed a tandem repeat structure at the *Rhg4* locus. A sequence of 35.7‐kb was duplicated at the *Rhg4* locus in ‘Peking’, PI 437654 and PI 438489B. The duplicated region contained four genes, including the cloned *Rhg4* gene, which encodes a serine hydroxymethyltransferase (SHMT). This discovery provides a new insight for the SCN resistance mechanism at the *Rhg4* locus.

During the last decade, many studies examined segmental duplication and genome re‐sequencing applications, with a special focus on the identification of CNVs (de Koning *et al*., 2011; Sharp *et al*., 2005; Zarrei *et al*., 2015; Lakhssassi *et al*., 2019). Deletions and duplications are considered to be major contributions to genome variability, playing important roles in generating variation among many traits, including disease phenotypes. Many studies explored the human genome for genetic disorders and identified a range of variants (Albertini *et al*., 1982; Inoue and Lupski, 2002; MacDonald *et al*., 1993; Myers, 2004; Perry *et al*., 2007) . However, CNV is an important type of structural variation because of its varied evolutionary impacts, stimulating genomic rearrangements and gene dosage effects (Flagel and Wendel, 2009; Moore and Purugganan, 2005; Olsen and Wendel, 2013). Different types of CNV have been observed in diverse organisms, including humans and chimpanzees (Perry *et al*., 2008), rats (Aitman *et al*., 2006), Arabidopsis (DeBolt, 2010), extremophile crucifer (Dassanayake *et al*., 2011) and *Plasmodium falciparum* (Heinberg *et al*., 2013). In soybean, it was reported that copy number of three genes collectively, at the *rhg1‐b* locus, encoding a soluble NSF‐attachment protein (α‐SNAP), an amino acid transporter, and a wound‐inducible domain (WI12), mediate nematode resistance in soybean PI 88788 type of resistance (Bayless *et al*., 2018; Cook *et al*., 2012). In this study, we provide a strong evidence that CNV of *GmSHMT08* at the *Rhg4* locus also plays a significant role in SCN resistance. Interestingly, mutations in human *SHMT* have been linked to a wide range of diseases (Lim *et al*., 2005; Maddocks *et al*., 2016; Skibola *et al*., 2002). An *shmt* knockout mutant was shown to induce apoptosis in lung cancer cells by causing uracil misincorporation (Paone *et al*., 2014). Therefore, the findings on SHMT allelic variation in this study may have implications beyond the field of plant pathology, as similar variants may be important within the field of pharmacogenomics due to SHMT's involvement in human cancer.

We demonstrated that the *Rhg4* resistant allele contains three critical spontaneously occurring natural mutation sites resulting in four amino acid changes; I37F (0.94%), P130R (15.1%), N358Y (11.32) and N358H (1.88%) at the GmSHMT08 protein when compared to the susceptible alleles. Homology modelling suggests that these point mutations may impair properties of the encoded GmSHMT08 enzyme, including subunit associations, PLP cofactor and substrate binding and catalytic site structure. The altered enzyme may further influence the folate homoeostasis in soybean root cells, and ultimately restrict the growth of cyst nematodes in susceptible soybean lines, as has been suggested previously (Liu *et al*., 2012). Our study demonstrated that the resistant *Rhg4* allele was detected in 13.2% of the sequenced soybean lines representing the USDA Soybean Germplasm Collection, including ‘Peking’. Additionally, it was reported that overexpression of *Rhg4*‐‘Peking’ in roots of SCN‐susceptible cultivar ‘Williams 82’ greatly reduced nematode parasitism (Matthews *et al*., 2013).

### Limited haplotypes and SCN resistance in the U.S. germplasm:

Since the discovery of SCN‐resistance QTL, most of the varieties in the U.S. trace back to ‘Peking’‐ and/or PI 88788‐type of resistance. Due to the effectiveness of the high copy *rhg1* from PI 88788 source, it was frequently utilized (over 95%) by breeders to develop elite cultivars (Bayless *et al*., 2016). However, limited variation, especially at the *Rhg4* locus was captured in the recent breeding programs. The effectiveness of PI 88788‐type resistance is breaking down due to continuous cropping of soybean varieties derived from PI 88788. However, due to virulence and adaptation of SCN populations, the high copy *rhg1* is not sufficient to confer broad‐based resistance unless epistatically interacting (additive) resistant haplotype are substituted (Meksem *et al*., 2001). The lack of genetic diversity and/or the right combination of resistant haplotypes has led to a widespread shift towards virulence in SCN populations (Gardner 2017; Lee *et al*., 2015). Our analysis showed that susceptibility phenotypes associated with low copies of *rhg1* could be overcome by incorporating *Rhg4* alleles.

The 106 WGRS set contains 57 elites, 44 landraces and seven wild soybean lines (Valliyodan *et al*., 2016). None of the elite lines carry multiple copies at the *Rhg4* locus and most of the lines (49/57) were highly susceptible to two or more SCN races (Table S4). To further confirm this result we utilized the whole‐genome sequence and CGH data from soybean NAM (Nested Association Mapping) population (Song *et al*., 2017) (https://www.soybase.org/SoyNAM/) and estimated CNV (Anderson *et al*., 2014) (Table S7). The soybean NAM populations consist of 17 high‐yielding lines from eight states from the U.S., 15 lines with diverse ancestry, eight lines are exotic PIs, in addition to the cv. ‘IA3023’, which was used as common parent for crossing with all 40 lines. Interestingly, eight out of 41 parents carry more than two copies of the *rhg1* locus with a maximum of 6.79 copies in LD02‐4485. However, at the *Rhg4* locus, no CNV was observed. This observation suggests that a limited number of resistant haplotypes were introgressed during past soybean breeding and variety development.

### Epistatic interactions between the *rhg1* and *Rhg4* loci

It has been reported that the interaction of two or more alleles (epistasis) plays a major role in an organism's resistance to diseases and pests (Bayless *et al*., 2016; Meksem *et al*., 2001; Nagel, 2005). The *rhg1* GmSNAP18 protein interacts with NSF (N‐ethylmaleimide–sensitive factor) protein and disturbs vesicle trafficking (Bayless *et al*., 2016, 2018). We discovered that soybean germplasm provided a wide range of SCN resistance controlled by natural variants (SNP and CNV) at both the *rhg1* and *Rhg4* loci. The CNV of the *rhg1* allele (2–10 copies) is a well‐known and documented resistance type (Bayless *et al*., 2016; Cook *et al*., 2014; Lee *et al*., 2015) that has been recently studied in more detail (Bayless *et al*., 2018). Epistasis between the *rhg1* and *Rhg4* has been studied in the past two decades (Brucker *et al*., 2005; Meksem *et al*., 2001). Recently, Yu *et al*. (2016) reported the advantage of the ‘Peking’‐type (an *Rhg4* required resistance) over the ‘Fayette’ (PI88788 type of resistance) and described the presence of a greatest resistance to two Hg‐types tested; Hg type 2.5.7 and Hg type 7, pointing to the *rhg1* copy number and type as important in determining the SCN resistance. In the previous study, the impact of the *Rhg4* on high copy *rhg1* soybean lines (PI88788 type) was not observed, suggesting that the difference between the *rhg1* sequences of ‘Peking’ and ‘Fayette’ might be the key to understanding the interaction between these alleles. In our study, we discovered the presence of the *Rhg4* copy number impacting SCN resistance. The *Rhg4‐a* ‘Peking’‐type *GmSHMT08* haplotype was not required for soybean lines carrying higher CN of the *rhg1* for both ‘Cloud’ and ‘PI88788’‐type of resistance, however, when the *rhg1* CN drops below the required threshold, the *Rhg4* ‘Peking’‐type is required to play a critical role for broad‐based resistance to SCN. We also revealed that the greatest resistance to both Hg‐types was an effect of the copy number of the *Rhg4‐a* ‘Peking’‐type. When a soybean line cumulated more than three copies of the *Rhg4‐a* ‘Peking’‐type, it gains broad‐based resistance to several Hg‐types.

The present study shows that all the 106‐soybean lines were grouped into six SCN resistance categories based on the genomic variation of *rhg1* and *Rhg4* loci (Table 1). Among these, 11 lines carrying 4.7–9.4 copies of *rhg1* mainly showed resistance to races 3 and 14, while 12 lines carrying both the ‘Peking’‐type of *rhg1‐a* and *Rhg4* (2.2–4.3 copies) showed greater resistance to races 1, 3 and 5 and were genotypically clustered. PI 437654 exhibited high resistance to multiple SCN races, including races 1, 2, 3, 5 and 14. Our analysis revealed that PI 437654 carries 3.3 copies of ‘Peking’‐type *rhg1‐a* and 4.3 copies of the ‘Peking’‐type *Rhg4*. Cultivar ‘Peking’ carries 3.2 copies of the ‘Peking’‐type *rhg1‐a* and 2.3 copies of ‘Peking’‐type *Rhg4*. It is likely that the CNV of the *Rhg4* gene impacts the difference in SCN resistance levels found between PI 437654 and ‘Peking’.

Among SCN‐resistant PIs characterized in this study, PI 407729, did not carry any known SCN resistance loci (*Rhg4 or rhg1*), but had resistance to multiple SCN races. This can be explained, in part, by the presence of the SNP in the *GmSHMT08* ^*+*^ promoter. These variations may correspond to transacting elements that can regulate other novel genes involved in SCN resistance beside the classic *rhg1* and *Rhg4* loci, and hence warrants further promoter analysis and gene functional characterization. Genetic mapping of the PI 407729 resistant‐QTL may reveal a previously unknown SCN resistance locus, conferring a unique mode of resistance. Results obtained from our study demonstrated that broad‐based resistance to multiple SCN races requires very specific haplotypes of the *rhg1* and *Rhg4* loci at the promoter, amino acid sequences and CNV. In fact, the type of interaction between the different alleles confers resistance to a given race that is haplotype‐dependent. This study shows that having more copies of *GmSHMT08* provides more transcript abundance, therefore reinforcing the resistance to SCN. Similar observations have been also revealed in the case of the *GmSNAP18* gene.

The genetic basis for broad‐based resistance to multiple races elucidated in this study will greatly benefit soybean breeders in the development of SCN‐resistance varieties. In addition, it will also help to select parental lines to design future crosses and trait introgressions. The SNP marker assays associated with CNV and SNP/indels can be used to stack multicopies of the *rhg1‐b* (PI88788‐type of resistance) or *Rhg4* (‘Peking’ type resistance) alleles for breeding purposes and will provide more sources for broad‐spectrum SCN resistance.

In summary, results obtained from our study reveal several new discoveries: (i) The *Rhg4* locus is a highly repeated region similar to the *rhg1* locus, likely consisting of a 35.7‐kb tandem repeat unit. Eleven lines with resistance to multiple races of SCN exhibited a CNV of 2.1–4.3 copies of *Rhg4* coupled with a ‘Peking’‐type *rhg1‐a* with copy numbers ranging from 1.9 to 3.5; (ii) the lines with PI 88788‐type *rhg1‐b* haplotypes required >5.6 copies to confer resistance to SCN races 3 and 14, regardless of the *Rhg4* haplotype; (iii) when *GmSNAP18* copy number dropped below 5.6 copies, a ‘Peking’‐type *GmSHMT08* haplotype was required to ensure resistance to SCN pointing to a novel mechanism of epistasis between the *GmSNAP18* and *GmSHMT08* involving minimum requirements for copy numbers at both loci; (iv) ‘Cloud’‐type *rhg1* performed better than ‘PI 88788’‐type *rhg1* and required less *GmSNAP18* copy numbers to confer SCN resistance; (v) when soybean lines accumulated more copies of the *GmSHMT08* gene, it acquired broad resistance to SCN; (vi) soybean lines with low CNV (1–3 copies) of ‘Peking’‐type *rhg1‐a* but lacked the *Rhg4* allele that showed resistance only to SCN race 5; (vii) both *rhg1* and *Rhg4* loci were in strong LD with the surrounding regions of the genome; (viii) expression analysis showed that transcript abundance of the *GmSHMT08* in root tissue correlated with more copies of the *Rhg4* locus, reinforcing the resistance to SCN; (ix) haplotype analysis of the *GmSHMT08* and *GmSNAP18* promoters provide an additional layer of the resistance mechanism. These findings can guide soybean breeders and provide them with new insight into epistatsis, haplotype compatibility, copy number variants, promoter variation and its impact on developing soybean lines with broad‐based disease resistance to SCN. The results suggest that breeders detect the alleles present and copy number of each as appropriate at both the *rhg1* and *Rhg4* loci for precise identification of the genetics of SCN resistance genotypes. A possible approach is that lines reported and released as resistant to SCN should be characterized at both the *rhg1* and *Rhg4* loci for CNV and source of each allele before being deployed as new SCN resistance germplasm.

## Material and methods

### Plant materials and SCN bioassays

One hundred and six soybean accessions and SCN indicator lines were evaluated for resistance to different HG Types of SCN. Homogenous nematode populations of races PA1 (HG Type 2.5.7), PA2 (HG Type 1.2.5.7), PA3 (HG Type 0), PA5 (HG Type 2.5.7) and PA14 (HG Type 1.3.5.6.7) have been maintained at the University of Missouri for more than 30 generations. SCN bioassays were performed in a greenhouse at the University of Missouri following a well‐established method (Arelli *et al*., 1997). Briefly, soybean seeds were germinated in paper pouches for 3–4 days and then transplanted into PVC tubes (100 cm^3^) (one plant per tube). The tubes were filled with steam‐pasteurized sandy soil and packed into plastic containers prior to transplanting. Each container held 25 tubes and was suspended over water baths maintained at 27 ± 1 °C. Five plants of each indicator line were arranged in a randomized complete block design. Two days after transplanting, each plant was inoculated with 2000 ± 25 SCN eggs. Thirty days post‐inoculation, nematode cysts were washed from the roots of each plant and counted using a fluorescence‐based imaging system (Brown *et al*., 2010). The female index (FI %) was estimated to evaluate the response of each plant to each race of SCN using the following formula: FI (%) = (average number of female cyst nematodes on a given individual/ average number of female nematodes on the susceptible check) × 100. Five replications were included for each tested lines and organized in randomized complete block design per experiment. Two independent experiments were performed and the final values are the averages of female index of two experiments. The FI values for all 106 lines are shown in Figure S1.

### Variant calling and haplotype analysis

The 106 soybean germplasm lines sequenced at approximately 17X genome coverage were utilized for mapping and detection of allelic variants (Valliyodan *et al*., 2016). The paired‐end re‐sequencing reads were mapped to the soybean reference genome, ‘Williams 82’ version 2 (W82.a2.v1.1) with BWA as described previously (Valliyodan *et al*., 2016; Zhou *et al*., 2015). SNP and Indels detection were performed using Genome Analysis Toolkit (GATK, V3.4.0) (McKenna *et al*., 2010) and SAMTools. For Indel calling, insertions and deletions shorter than or equal to 6 bp were taken into consideration. CNV was detected according to depth distribution of each line (Zhou *et al*., 2015). Regions were regarded as CNVs if their minimum length was >2‐kb and their mean depth was less than half of the sequence depth or more than double of the sequence depth. The initial and final minimum probability to merge the adjacent breakpoint was set to 0.5 and 0.8 respectively. Additionally, CNV of indicator lines was visualized using GenomeBrowse (http://goldenhelix.com/). Haplotype analysis of the *rhg1* and *Rhg4* loci was performed using a pipeline as previously described by Patil *et al*. (2016). Briefly, SNP haplotypes were examined by generating map and genotype data files and clustering pictorial output for the *rhg1* and *Rhg4* genomic regions were visualized using FLAPJACK (Milne *et al*., 2010). The SNP identified from each line were clustered based on neighbour‐joining (NJ) tree output and SNPs were further analysed for possible synonymous/non‐synonymous variation by translation into amino acid sequences. The SNP diversity, average pairwise divergence within population (θ_π_), Watterson's estimator (θ_*w*_) and *F*_st_ were estimated as previously described (Valliyodan *et al*., 2016).

### Comparative genomic hybridizations, Taqman assays and digital PCR

Comparative genomic hybridizations assay was adapted as described by McHale *et al*. (2012) and Dobbels *et al*. (2017). The Taqman assay was performed according to Kadam *et al*. (2016). The digital PCR was performed according to Wan *et al*. (2016). Briefly, 20 μL reaction was prepared, consisting of 10 μL 2× master reaction mix (Life Technologies, Waltham, MA), 1 μL assay mix (18 μm Forward and 18 μm reverse primers + 5 μm probe), 1 μL DNA (final concentration 40 ng) and 9 μL ddH2O. The forward and reverse probe/primer sequences were used according to Kadam *et al*. (2016). A 14.5 μL of the PCR mixture was loaded onto a QuantStudio™ 3D Digital PCR 20K Chip (Thermo Fisher Scientific, Waltham, MA). The chip was covered with immersion fluid, a lid was applied, the assembly was filled with immersion fluid and the loading port was sealed according to the manufacturer's instructions. The chips were loaded into the Dual Flat Block GeneAmpR PCR System 9700 (Life Technologies), and PCR was performed using the following conditions: 96 °C for 10 min; 60 °C for 2 min and 98 °C for 30 s, for 39 cycles; 60 °C for 2 min; 10 °C for storage. The Digital PCR 20K Chip was read using the QuantStudio™ 3D Digital PCR Chip Reader, and the data was analysed using the QuantStudio™ 3D AnalysisSuite™ Software (Thermo Fisher Scientific, Waltham, MA).

### Identification of tandem repeats at the *Rhg4* locus

Aliquots of the genomic DNA samples isolated for whole genome re‐sequencing were used in PCR reactions. The PCR reactions were conducted using PrimeSTAR GXL DNA Polymerase from Takara Bio USA, Inc., according to the manufacturer's instructions (Takara Bio USA, Inc., formerly known as Clontech Laboratories, Mountain View, CA).

### Protein homology modelling of *GmSNAP18* and *GmSHMT08* and interaction analysis

Homology modelling of a putative GmSNAP18 and GmSHMT08 protein structure was conducted as previously described (Lakhssassi *et al*., 2019; Liu *et al*., 2017). To induce and map the corresponding existing natural mutations (haplotypes) between the susceptible and resistant soybeans lines of the GmSHMT08 protein, the structural editing tool from UCSF Chimera package was employed. Additionally, the impact of catalytic activity of the enzyme homodimerization, tetramerization and/or substrate binding was studied. Approximately 5.0 Å containing all atoms/bonds of any residue surrounding the mutated residue was selected first and shown in the model to study all possible residue interactions. Next, the rotamers tool was used to mutate the three residues (I37F, Y358N/H and R130P) and to predict residue interactions and possible impact on protein activity and/or structure.

### qRT‐PCR of *GmSNAP18* and *GmSHMT08* genes

Three‐day old soybean seedlings of different indicator lines were germinated and inoculated with freshly hatched second‐stage juveniles of SCN race PA3, PA5 and PA14 as previously described by Rambani *et al*. (2015). Three biological samples of inoculated and noninoculated root tissues were collected at 2 days’ post‐inoculation and used for RNA extraction and qPCR analysis. Total RNA was isolated using Qiagen RNeasy Plant Mini Kit (cat# 74904) from root samples collected 2 days after SCN infection. Total RNA was DNase treated and purified using Turbo DNA‐free Kit (QAmbion/Life Technologies AM1907, Waltham, MA USA). RNA was quantified using Nanodrop 1000 (V3.7), then a total of 400 ng of treated RNA was used to generate cDNA using the cDNA synthesis Kit (Thermoscript, Life Technologies, #11146‐025), with random hexamers. About 1/10th of a 20 μL reverse transcription reaction was used in gene‐specific qPCR with the Power SYBR^®^ Green PCR Master Mix Kit (Applied Biosystems™ #4368706, Grand Island, NY, USA). Primers used in this study were described previously (Rambani *et al*., 2015). For qRT‐PCR, the *Ubiquitin‐3* (Glyma20g27950) gene has been used as endogenous control as previously described by Liu *et al*. (2017). The PCR products for soybean *Ubiquitin‐3* (*GmUBI‐3*) were used to judge equality of concentration of cDNA templates in different samples. PCR efficiencies for target (*GmSHMT08* and *GmSNAP18*) and reference (*GmUBI‐3*) genes were equal among samples. The qRT‐PCR data were analysed as previously described by (Lakhssassi *et al*., 2017b).

## Authors contributions

GBP conducted the WGRS, copy number, haplotype analysis, epistasis interaction, data interpretation and manuscript writing. NL performed structure‐based homology modelling, mutational analysis, qRT‐PCR analysis, CN and haplotype data interpretation and manuscript writing. JW and LS performed digital PCR and Taqman assay. TDV designed SCN phenotyping experiment and LS and MK assisted with greenhouse phenotyping. AOS performed CGH analysis. BV provided whole genome sequence data. VC did homology modelling. SSK, ZZ, HJR, SP performed SCN infection, tissue collection and RNA extractions. TH facilitated SCN infection for expression analysis and edited the manuscript. RMS facilitated and provided CGH data for 106 and NAM parent lines and performed data interpretation and manuscript editing. KM and HTN conceived the study, performed data interpretation and edited the manuscript. All authors read and approved the final manuscript.

## Competing interest

The authors declare they have no competing interests.

## Ethics and consent to participate

This study did not involve humans, human data or animals; no ethics approval or consent is required to publish the results.

## Availability of data and materials

All generated data and material can be accessed at any time. Access to some material is subject to Material Transfer Agreement (MTA).

## Supporting information


**Figure S1** Female index for SCN Race 1, 2, 3, 5 and 14 from the 106 soybean lines in the present study.
**Figure S2** Diversity, linkage disequilibrium (LD) and sequence analysis of region surrounding the *Rhg1* and *Rhg4* loci.
**Figure S3** Copy number variation (CNV) of the (A) Rhg1 and (B) Rhg4 locus defined from whole‐genome re‐sequencing for SCN‐resistant lines.
**Figure S4** Graphical representation of CNV using whole genome sequencing data.
**Figure S5** Copy number variation (CNV) of the *Rhg1* (A) and *Rhg4* (B) loci were validated using a comparative genomic hybridization (CGH) method.
**Figure S6** PCR amplification of the regions surrounding Glyma.08g108900 (Rhg4) in different soybean lines.
**Figure S7** Graphical illustrations of the strategies employed to obtain the junction regions between two neighbouring repeats.
**Figure S8** Confirmation of the junction regions between two neighbouring repeats in different soybean lines.
**Figure S9** The identified repeat at the Rhg4 locus.

**Table S1** Statistics of DNA variant analysis for *rhg1* from SCN‐resistant lines.
**Table S2** Statistics for DNA variant analysis of the *rhg1* and *Rhg4* loci from SCN‐resistant lines.
**Table S3** Primers used to study the *Rhg4* duplication.
**Table S4** Summary of haplotype clusters, reaction to SCN races, CNV and type of *rhg1* and *Rhg4* resistance lines.
**Table S5** Requirement of *rhg1* and *Rhg4* copies in the presence and absence of GmSHMT08 promoter to confer SCN resistance.
**Table S6** Female index of soybean accessions used for gene expression analysis against five soybean cyst nematode populations: Race 1 (HG Type 2.5.7), Race 2 (HG Type 1.2.5.7), Race 3 (HG Type 0), Race 5 (HG Type 2.5.7) and Race 14 (HG Type 1.3.6.7).
**Table S7** Estimation of CNV using whole‐genome sequence and comparative genome hybridization in NAM population.
