# Supplementary material for: Whole‐genome re‐sequencing reveals the impact of the interaction of copy number variants of the rhg1 and Rhg4 genes on broad‐based resistance to soybean cyst nematode
Source: Plant Biotechnol J. 2019 Feb 20;17(8):1595–611. doi: 10.1111/pbi.13086 (PMC6662113; doi:10.1111/pbi.13086)
Supplement: Supplementary file 1 — Figure S1 Female index for SCN Race 1, 2, 3, 5 and 14 from the 106 soybean lines in the present study. Figure S2 Diversity, linkage disequilibrium (LD) and sequence analysis of region surrounding the Rhg1 and Rhg4 loci. Figure S3 Copy number variation (CNV) of the (A) Rhg1 and (B) Rhg4 locus defined from whole‐genome re‐sequencing for SCN‐resistant lines. Figure S4 Graphical representation of CNV using whole genome sequencing data. Figure S5 Copy number variation (CNV) of the Rhg1 (A) and Rhg4 (B) loci were validated using a comparative genomic hybridization (CGH) method. Figure S6 PCR amplification of the regions surrounding Glyma.08g108900 (Rhg4) in different soybean lines. Figure S7 Graphical illustrations of the strategies employed to obtain the junction regions between two neighbouring repeats. Figure S8 Confirmation of the junction regions between two neighbouring repeats in different soybean lines. Figure S9 The identified repeat at the Rhg4 locus. [file PBI-17-1595-s002.pptx]

## Slide 1
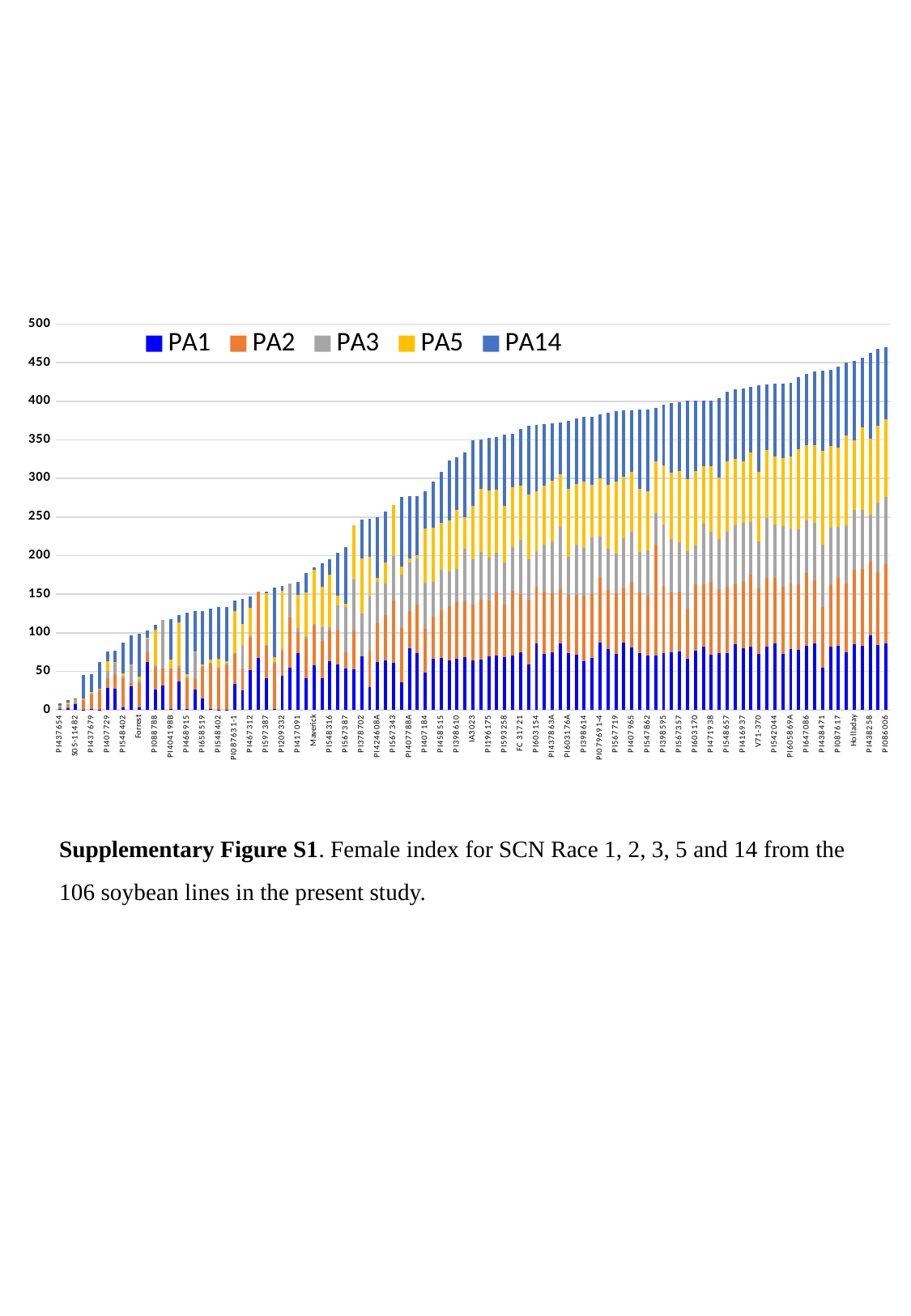

### Chart
| Category | PA1 | PA2 | PA3 | PA5 | PA14 |
|---|---|---|---|---|---|
| PI437654 | 0.841121495327103 | 2.0 | 1.0 | 2.0 | 3.0 |
| PI090763 | 2.0 | 4.0 | 2.0 | 2.0 | 3.0 |
| S05-11482 | 7.663551401869157 | 0.70635721493441 | 4.337050805452291 | 0.920598388952819 | 0.965250965250965 |
| PI089772 | 0.654205607476636 | 12.20988900100908 | 0.743494423791822 | 1.035673187571922 | 30.79150579150579 |
| PI437679 | 0.841121495327103 | 18.86982845610494 | 1.363073110285006 | 2.186421173762946 | 22.77992277992278 |
| PI404166 | 0.654205607476636 | 24.52068617558022 | 1.363073110285006 | 1.035673187571922 | 34.16988416988416 |
| PI407729 | 28.0 | 13.0 | 10.0 | 12.0 | 13.0 |
| PI567516C | 27.196261682243 | 18.16347124117053 | 15.36555142503098 | 1.611047180667434 | 14.76833976833977 |
| PI548402 | 3.0 | 38.0 | 2.0 | 4.0 | 40.0 |
| PI567305 | 30.46728971962617 | 4.641775983854692 | 22.30483271375465 | 1.150747986191024 | 37.54826254826254 |
| Forrest | 3.0 | 32.0 | 3.0 | 5.0 | 56.0 |
| S10-11227 | 62.2429906542056 | 12.9162462159435 | 16.23296158612143 | 2.531645569620253 | 9.45945945945946 |
| PI088788 | 26.0 | 29.0 | 2.47831474597274 | 46.0 | 7.0 |
| PI424088 | 32.0 | 22.0 | 63.0 | 0.0 | 0.0 |
| PI404198B | 0.934579439252336 | 51.33703329969727 | 1.363073110285006 | 11.73762945914845 | 52.5096525096525 |
| PI495017C | 36.91588785046727 | 17.05348133198789 | 2.973977695167286 | 56.73187571921748 | 9.330759330759333 |
| PI468915 | 1.121495327102804 | 39.73259334006056 | 1.084262701363073 | 4.602991944764096 | 79.53667953667953 |
| PI567336B | 26.6355140186916 | 13.6226034308779 | 34.44857496902107 | 1.035673187571922 | 51.83397683397684 |
| PI658519 | 15.32710280373832 | 40.36326942482341 | 1.115241635687732 | 2.301495972382048 | 69.40154440154441 |
| PI437690 | 1.02803738317757 | 58.52674066599395 | 1.734820322180917 | 3.567318757192175 | 66.89189189189189 |
| PI548402 | 0.654205607476636 | 53.2795156407669 | 1.239157372986369 | 11.50747986191024 | 66.60231660231659 |
| PI437725 | 0.373831775700935 | 57.71947527749747 | 1.734820322180917 | 3.222094361334867 | 70.46332046332049 |
| PI087631-1 | 34.11214953271028 | 38.34510595358224 | 0.991325898389096 | 55.12082853855005 | 13.03088803088803 |
| PI548317 | 25.23364485981308 | 28.25428859737639 | 30.48327137546468 | 27.27272727272726 | 32.81853281853282 |
| PI467312 | 51.96261682242992 | 41.47325933400606 | 2.106567534076828 | 36.93901035673188 | 14.72007722007722 |
| PI424078 | 66.93925233644858 | 86.0242179616549 | 0.0 | 0.0 | 0.0 |
| PI597387 | 41.21495327102804 | 42.68415741675076 | 0.619578686493185 | 66.39815880322209 | 2.606177606177607 |
| PI507354 | 1.495327102803738 | 58.02219979818367 | 2.47831474597274 | 6.444188722669734 | 90.37162162162163 |
| PI209332 | 44.39252336448598 | 32.39152371342079 | 1.61090458488228 | 76.1795166858458 | 5.952380952380952 |
| PI567230 | 55.0467289719626 | 64.48032290615538 | 44.23791821561338 | 0.0 | 0.0 |
| PI417091 | 73.83177570093456 | 26.63975782038345 | 5.328376703841387 | 42.80782508630609 | 17.47104247104247 |
| PI639740 | 40.65420560747663 | 50.3531786074672 | 3.469640644361834 | 57.53739930955121 | 25.57915057915058 |
| Maverick | 57.38317757009344 | 51.3622603430878 | 2.230483271375465 | 70.65592635212884 | 3.378378378378378 |
| PI548349 | 40.5607476635514 | 48.23410696266397 | 17.84386617100372 | 52.47410817031069 | 31.27413127413126 |
| PI548316 | 63.17757009345794 | 39.9596367305752 | 3.717472118959108 | 68.46950517836594 | 20.07722007722008 |
| PI612611 | 59.25233644859813 | 45.20686175580221 | 30.85501858736059 | 13.0897583429229 | 55.5019305019305 |
| PI567387 | 53.55140186915887 | 21.44298688193743 | 58.85997521685253 | 4.142692750287687 | 72.87644787644786 |
| PI407162 | 52.64797507788163 | 50.07568113017155 | 65.79925650557622 | 70.65592635212884 | 0.0 |
| PI378702 | 68.92523364485982 | 0.0 | 56.5365551425031 | 70.33947065592635 | 51.31917631917632 |
| PI366121 | 29.32242990654206 | 46.41775983854692 | 70.83849648905411 | 52.21518987341772 | 48.503861003861 |
| PI424608A | 62.05607476635513 | 50.15136226034308 | 53.53159851301115 | 4.948216340621403 | 79.63320463320464 |
| PI549031 | 63.73831775700934 | 59.23309788092836 | 41.01610904584882 | 27.38780207134637 | 65.34749034749035 |
| PI567343 | 60.84112149532709 | 80.42381432896065 | 58.44692275919041 | 65.47756041426926 | 0.0 |
| PI424298 | 36.26168224299065 | 70.23208879919274 | 68.4014869888476 | 10.70195627157652 | 90.92664092664093 |
| PI407788A | 79.81308411214953 | 48.13319878910193 | 63.56877323420074 | 5.293440736478709 | 80.1158301158301 |
| PI603497 | 73.45794392523364 | 62.86579212916246 | 59.97521685254027 | 4.602991944764096 | 76.15830115830114 |
| PI407184 | 48.75389408099688 | 56.00403632694248 | 59.97521685254027 | 70.38741848868429 | 47.97297297297298 |
| PI548415 | 66.35514018691589 | 53.9858728557013 | 45.60099132589838 | 70.54085155350977 | 59.84555984555986 |
| PI458515 | 67.196261682243 | 62.46215943491424 | 52.2924411400248 | 60.98964326812426 | 65.73359073359072 |
| PI464920B | 64.29906542056074 | 69.92936427850655 | 45.72490706319702 | 65.24741081703105 | 78.28185328185327 |
| PI398610 | 66.4485981308411 | 73.36024217961655 | 43.12267657992565 | 76.2945914844649 | 67.85714285714285 |
| PI567519 | 68.3177570093458 | 72.65388496468215 | 67.78190830235437 | 41.19677790563865 | 83.88030888030889 |
| IA3023 | 63.92523364485982 | 72.75479313824418 | 58.24039653035935 | 69.62025316455694 | 84.55598455598454 |
| S07-5049 | 65.42056074766354 | 77.49747729566096 | 62.08178438661709 | 81.81818181818177 | 63.22393822393824 |
| PI196175 | 69.34579439252335 | 72.75479313824418 | 55.26641883519206 | 86.65132336018411 | 68.91891891891893 |
| PI398593 | 70.3271028037383 | 82.2401614530777 | 51.42503097893433 | 81.70310701956271 | 68.14671814671813 |
| PI593258 | 68.0 | 69.0 | 54.0 | 73.0 | 93.0 |
| PI567354 | 70.5607476635514 | 84.25832492431888 | 56.62949194547707 | 76.66858457997698 | 69.30501930501931 |
| FC 31721 | 74.53271028037383 | 75.68113017154388 | 70.13630731102847 | 70.19562715765244 | 73.9623552123552 |
| PI518751 | 58.69158878504671 | 84.56104944500505 | 51.54894671623295 | 83.86075949367088 | 89.1650579150579 |
| PI603154 | 85.98130841121495 | 73.76387487386476 | 45.47707558859975 | 78.48101265822785 | 85.74646074646076 |
| PI567611 | 72.71028037383175 | 79.51564076690212 | 61.83395291201982 | 76.98504027617952 | 79.53667953667953 |
| PI437863A | 74.4859813084112 | 76.3874873864783 | 67.16232961586121 | 78.48101265822785 | 75.19305019305018 |
| PI561271 | 85.98130841121495 | 70.0 | 81.0 | 68.0 | 67.0 |
| PI603176A | 74.11214953271026 | 74.67204843592329 | 49.93804213135068 | 87.34177215189872 | 88.64221364221368 |
| PI591539 | 71.9626168224299 | 77.29566094853682 | 64.68401486988847 | 78.94131185270423 | 84.36293436293435 |
| PI398614 | 63.27102803738318 | 85.1664984863774 | 61.33828996282528 | 86.1910241657077 | 83.97683397683397 |
| PI548667 | 67.0 | 83.0 | 74.0 | 68.0 | 88.0 |
| PI079691-4 | 87.10280373831776 | 83.75378405650856 | 54.21313506815365 | 75.23014959723822 | 82.40830115830116 |
| PI518664 | 78.69158878504673 | 76.48839556004037 | 54.02726146220569 | 82.62370540851552 | 93.53281853281852 |
| PI567719 | 72.54672897196262 | 78.70837537840563 | 51.57992565055763 | 92.77905638665129 | 91.69884169884169 |
| PI567731 | 87.47663551401868 | 70.53481331987892 | 64.1883519206939 | 79.51668584579976 | 86.29343629343629 |
| PI407965 | 80.93457943925235 | 84.56104944500505 | 65.30359355638166 | 77.79056386651322 | 79.72972972972971 |
| PI437169B | 73.6448598130841 | 78.1029263370333 | 52.6641883519207 | 81.81818181818177 | 103.0888030888031 |
| PI547862 | 70.0 | 77.9011099899092 | 58.85997521685253 | 76.52474108170311 | 106.4671814671815 |
| PI567383 | 70.0 | 144.6 | 40.272614622057 | 67.31875719217487 | 69.40154440154441 |
| PI398595 | 73.9485981308411 | 87.0332996972755 | 79.80173482032218 | 75.56578442654391 | 78.66795366795368 |
| PI552538 | 74.39252336448595 | 77.80020181634711 | 69.14498141263937 | 86.4211737629459 | 89.57528957528956 |
| PI567357 | 75.88785046728971 | 77.80020181634711 | 64.06443618339527 | 91.94476409666284 | 88.6100386100386 |
| PI548511 | 66.19937694704048 | 64.88395560040361 | 74.34944237918215 | 93.67088607594935 | 101.4720077220077 |
| PI603170 | 76.91588785046727 | 85.36831483350149 | 51.0532837670384 | 96.31760644418873 | 90.92664092664093 |
| Reference | 82.0 | 81.0 | 78.0 | 75.0 | 85.0 |
| PI471938 | 71.30841121495324 | 94.3491422805247 | 65.55142503097893 | 84.34982738780207 | 85.52123552123552 |
| PI417015 | 73.83177570093456 | 82.84561049445006 | 65.05576208178437 | 79.63176064441888 | 102.6061776061776 |
| PI548657 | 73.36448598130843 | 85.77194752774973 | 72.11895910780669 | 90.44879171461447 | 90.4440154440155 |
| PI567690 | 85.14018691588785 | 77.19475277497476 | 76.45600991325898 | 86.3060989643268 | 90.83011583011583 |
| PI416937 | 79.90654205607476 | 86.5792129162462 | 75.83643122676577 | 80.09205983889525 | 94.2326254826255 |
| PI594512A | 81.77570093457942 | 93.7436932391524 | 67.78190830235437 | 90.7940161104718 | 84.74903474903476 |
| V71-370 | 72.24299065420561 | 84.76286579212916 | 61.80297397769515 | 89.29804372842345 | 112.5482625482626 |
| PI567651 | 82.2429906542056 | 89.1019172552977 | 77.69516728624536 | 87.7445339470656 | 85.03861003861005 |
| PI542044 | 85.88785046728971 | 84.86377396569121 | 69.14498141263937 | 89.06789413118527 | 93.33976833976833 |
| PI594012 | 72.05607476635514 | 87.48738647830476 | 78.19083023543988 | 88.37744533947064 | 96.52509652509652 |
| PI605869A | 79.15887850467287 | 84.66195761856711 | 71.1276332094176 | 93.44073647871117 | 94.980694980695 |
| PI408105A | 77.92056074766354 | 84.96468213925328 | 70.75588599752167 | 104.1426927502877 | 93.53281853281852 |
| PI647086 | 83.27102803738316 | 94.14732593340061 | 68.15365551425032 | 97.46835443037976 | 92.1814671814672 |
| PI603175 | 85.79439252336448 | 82.2401614530777 | 74.10161090458486 | 101.4959723820483 | 95.36679536679536 |
| PI438471 | 55.14018691588785 | 78.1029263370333 | 80.39033457249067 | 121.8642117376295 | 103.8610038610039 |
| PI248515 | 81.58878504672894 | 80.62563067608474 | 74.34944237918215 | 105.7537399309551 | 98.55212355212353 |
| PI087617 | 83.08411214953271 | 87.89101917255296 | 66.0470879801735 | 102.6467203682393 | 105.0193050193051 |
| PI594599 | 74.9221183800623 | 89.30373360242177 | 75.21685254027263 | 116.1104718066743 | 94.30501930501931 |
| Holladay | 85.04672897196262 | 96.56912209889 | 77.32342007434944 | 90.44879171461447 | 102.7992277992278 |
| PI475783B | 83.3644859813084 | 98.78910191725531 | 76.82775712515487 | 106.6743383199079 | 90.25096525096527 |
| PI438258 | 96.26168224299067 | 95.86276488395559 | 61.18339529120198 | 98.61910241657075 | 110.8429858429858 |
| PI200508 | 83.55140186915888 | 95.35822401614531 | 89.83890954151177 | 100.0 | 99.03474903474904 |
| PI086006 | 85.79439252336448 | 103.531786074672 | 86.24535315985128 | 101.150747986191 | 93.33976833976833 |Supplementary Figure S1. Female index for SCN Race 1, 2, 3, 5 and 14 from the 106 soybean lines in the present study.

## Slide 2
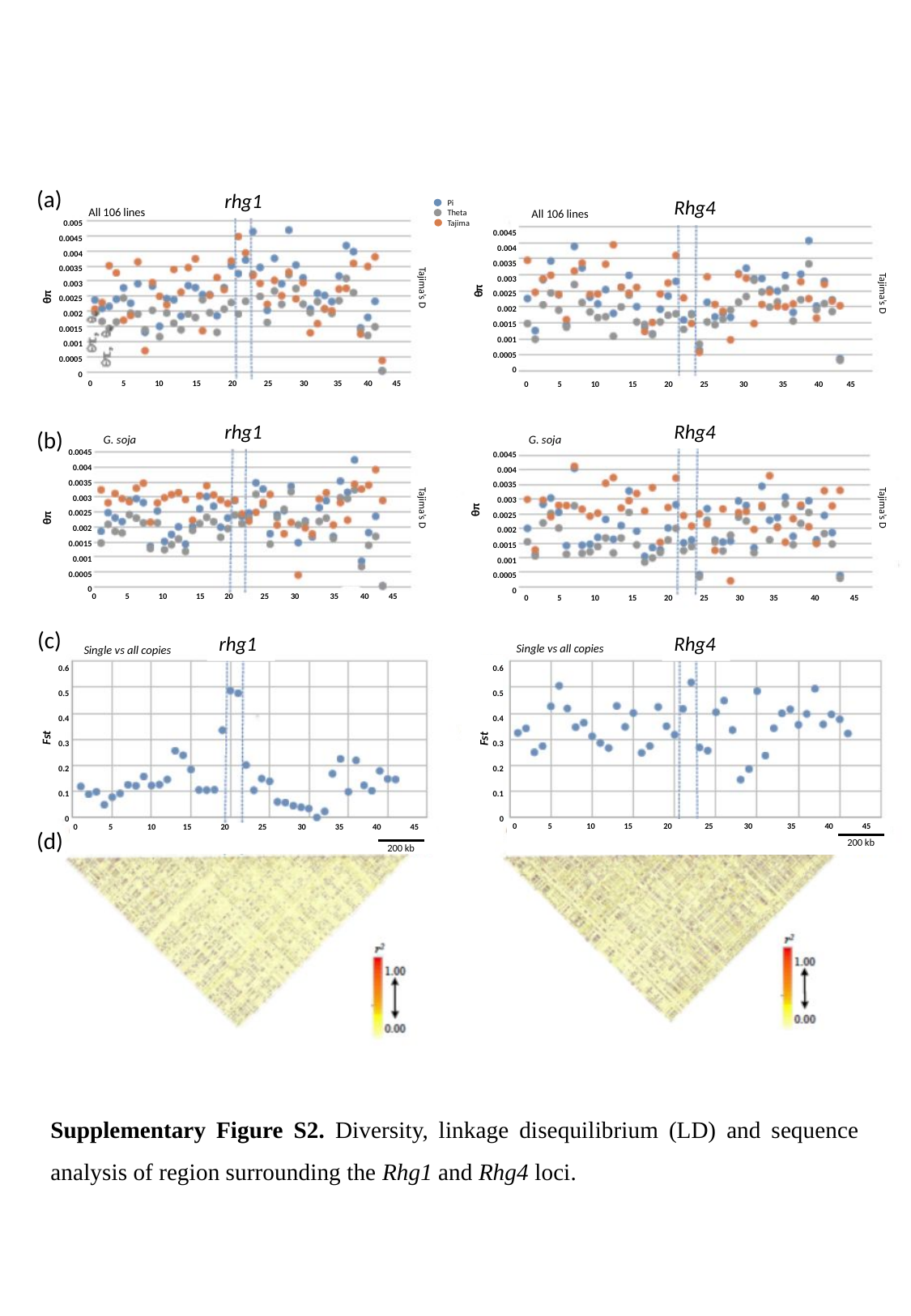

(a)
rhg1
Rhg4
Pi
Theta
Tajima
All 106 lines
All 106 lines
0.005
0.0045
0.004
0.0035
0.003
0.0025
0.002
0.0015
0.001
0.0005
0
0.0045
0.004
0.0035
0.003
0.0025
0.002
0.0015
0.001
0.0005
0
Tajima’s D
θπ
Tajima’s D
θπ
0 5 10 15 20 25 30 35 40 45
0 5 10 15 20 25 30 35 40 45
Rhg4
rhg1
(b)
G. soja
G. soja
0.0045
0.004
0.0035
0.003
0.0025
0.002
0.0015
0.001
0.0005
0
0.0045
0.004
0.0035
0.003
0.0025
0.002
0.0015
0.001
0.0005
0
θπ
Tajima’s D
Tajima’s D
θπ
0 5 10 15 20 25 30 35 40 45
0 5 10 15 20 25 30 35 40 45
(c)
Rhg4
rhg1
Single vs all copies
Single vs all copies
0.6
0.5
0.4
0.3
0.2
0.1
0
0.6
0.5
0.4
0.3
0.2
0.1
0
Fst
Fst
0 5 10 15 20 25 30 35 40 45
0 5 10 15 20 25 30 35 40 45
(d)
200 kb
200 kb
Supplementary Figure S2. Diversity, linkage disequilibrium (LD) and sequence analysis of region surrounding the Rhg1 and Rhg4 loci.

## Slide 3
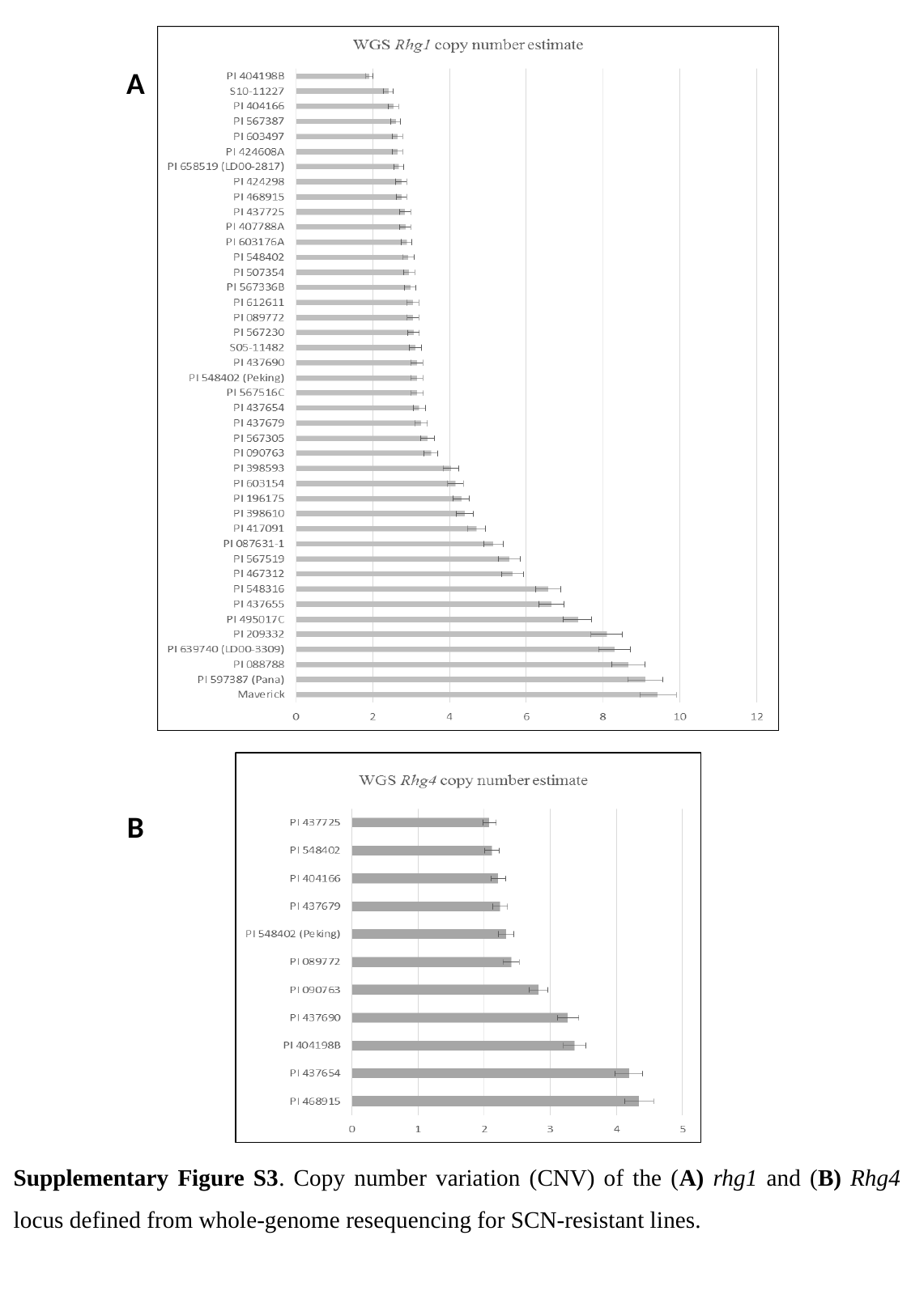

A
B
Supplementary Figure S3. Copy number variation (CNV) of the (A) rhg1 and (B) Rhg4 locus defined from whole-genome resequencing for SCN-resistant lines.

## Slide 4
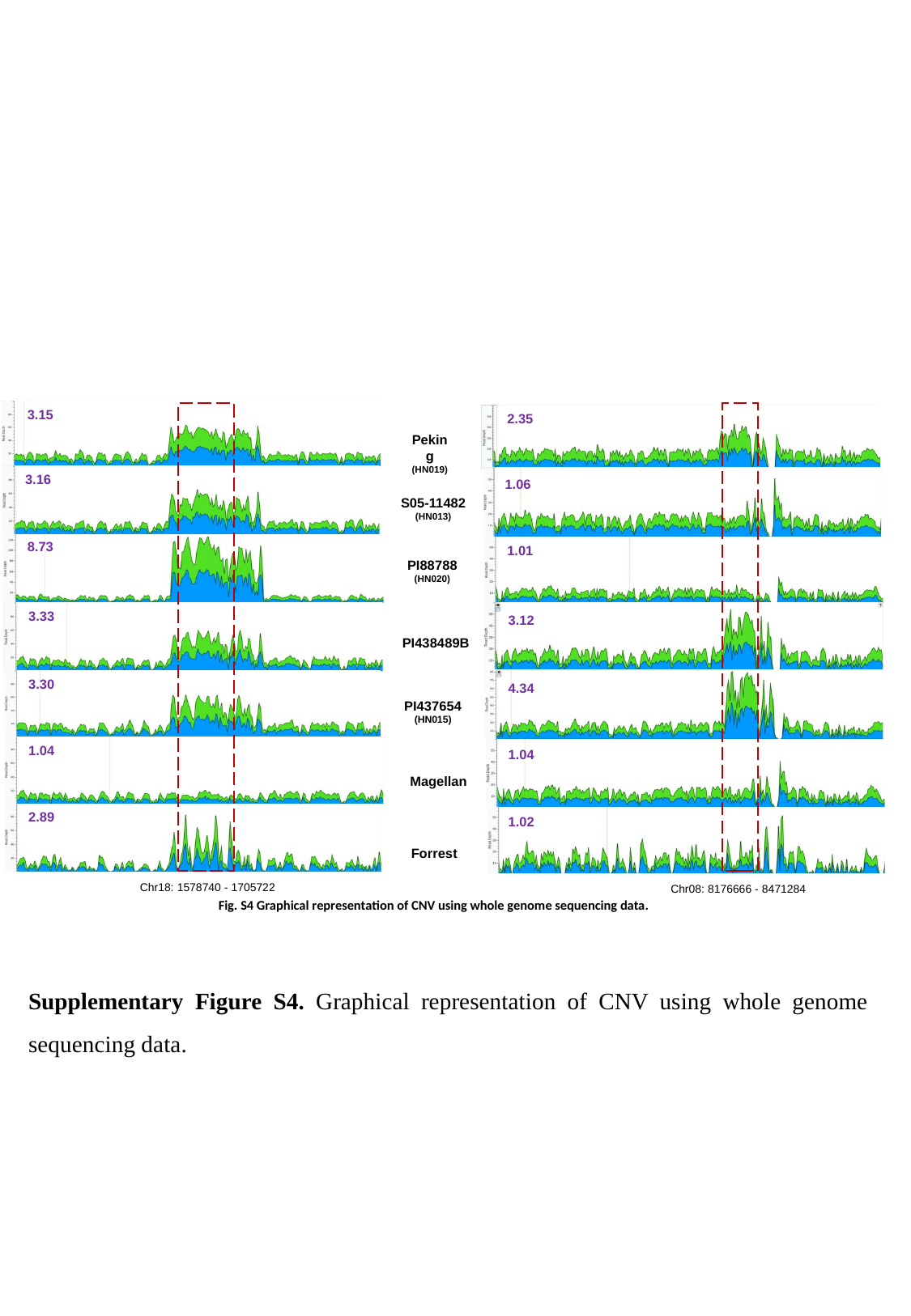

3.15
Chr08: 8176666 - 8471284
2.35
Peking
(HN019)
3.16
1.06
S05-11482
(HN013)
8.73
1.01
PI88788
(HN020)
3.33
3.12
PI438489B
3.30
4.34
PI437654
(HN015)
1.04
1.04
Magellan
2.89
1.02
Forrest
Chr18: 1578740 - 1705722
Chr08: 8176666 - 8471284
Fig. S4 Graphical representation of CNV using whole genome sequencing data.
Supplementary Figure S4. Graphical representation of CNV using whole genome sequencing data.

## Slide 5
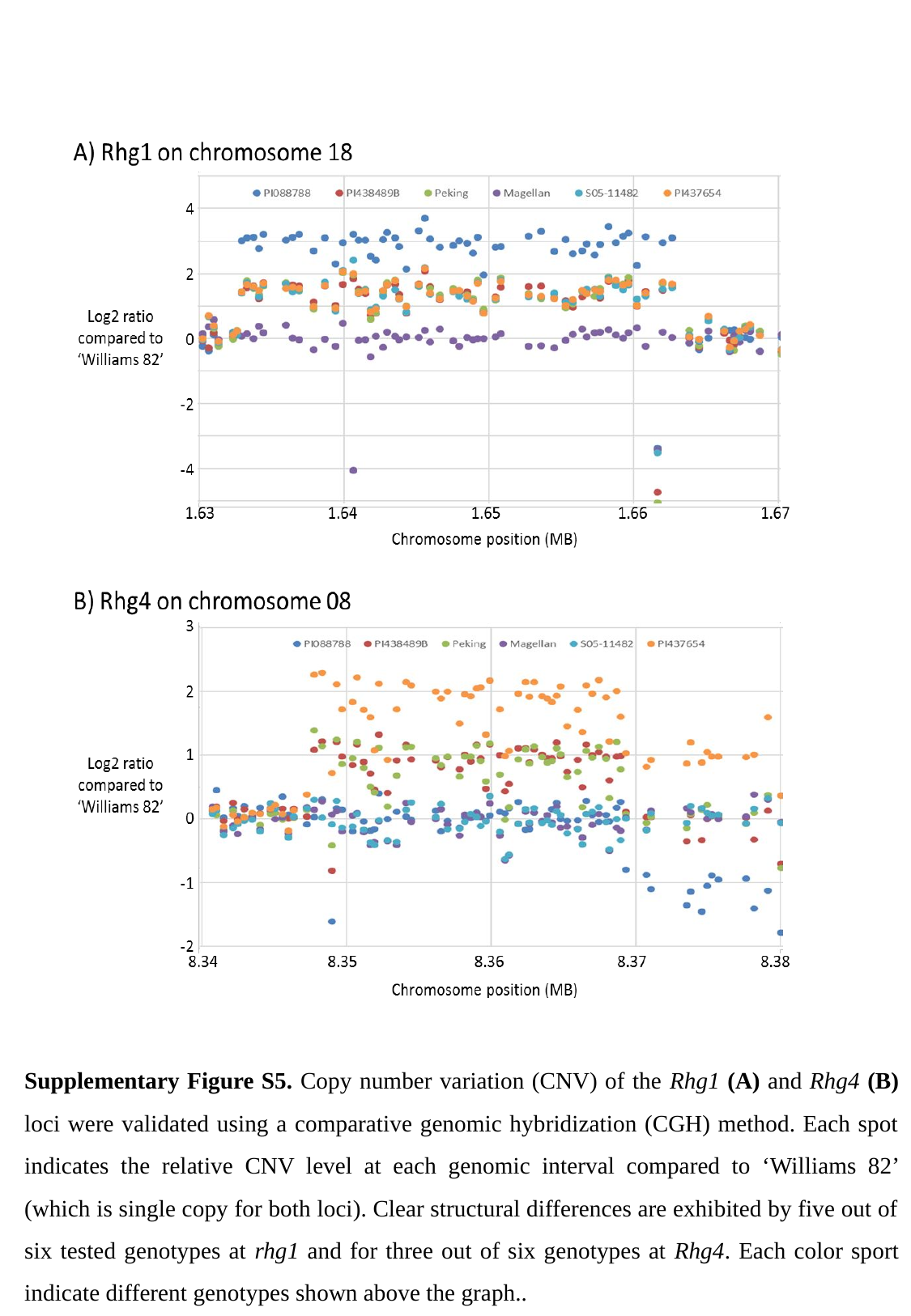

Supplementary Figure S5. Copy number variation (CNV) of the Rhg1 (A) and Rhg4 (B) loci were validated using a comparative genomic hybridization (CGH) method. Each spot indicates the relative CNV level at each genomic interval compared to ‘Williams 82’ (which is single copy for both loci). Clear structural differences are exhibited by five out of six tested genotypes at rhg1 and for three out of six genotypes at Rhg4. Each color sport indicate different genotypes shown above the graph..

## Slide 6
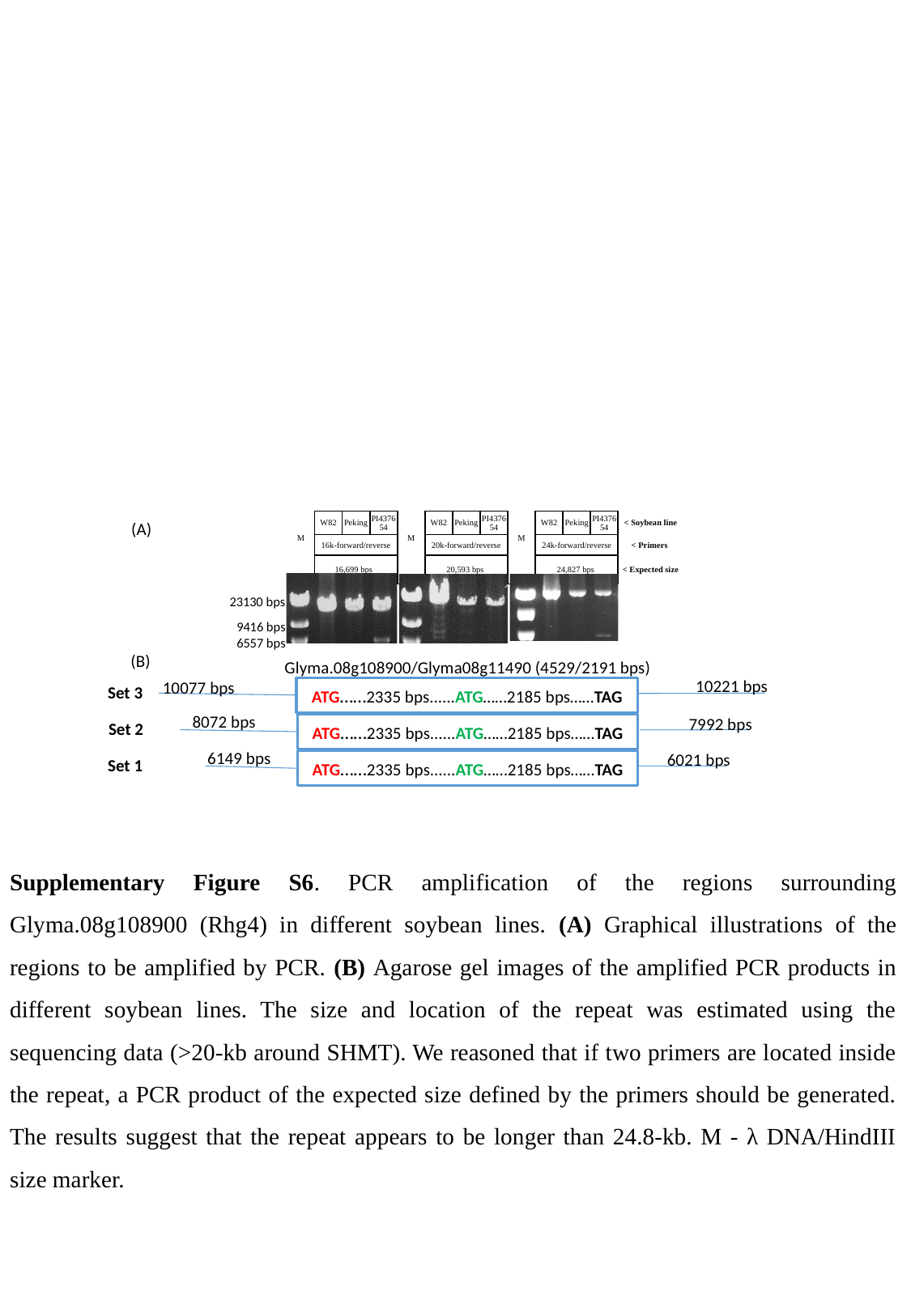

| M | W82 | Peking | PI437654 | M | W82 | Peking | PI437654 | M | W82 | Peking | PI437654 | < Soybean line |
| --- | --- | --- | --- | --- | --- | --- | --- | --- | --- | --- | --- | --- |
| | 16k-forward/reverse | | | | 20k-forward/reverse | | | | 24k-forward/reverse | | | < Primers |
| | 16,699 bps | | | | 20,593 bps | | | | 24,827 bps | | | < Expected size |
(A)
23130 bps
9416 bps
6557 bps
(B)
Glyma.08g108900/Glyma08g11490 (4529/2191 bps)
10221 bps
10077 bps
Set 3
ATG……2335 bps......ATG……2185 bps……TAG
8072 bps
7992 bps
Set 2
ATG……2335 bps......ATG……2185 bps……TAG
6149 bps
6021 bps
Set 1
ATG……2335 bps......ATG……2185 bps……TAG
Supplementary Figure S6. PCR amplification of the regions surrounding Glyma.08g108900 (Rhg4) in different soybean lines. (A) Graphical illustrations of the regions to be amplified by PCR. (B) Agarose gel images of the amplified PCR products in different soybean lines. The size and location of the repeat was estimated using the sequencing data (>20-kb around SHMT). We reasoned that if two primers are located inside the repeat, a PCR product of the expected size defined by the primers should be generated. The results suggest that the repeat appears to be longer than 24.8-kb. M - λ DNA/HindIII size marker.

## Slide 7
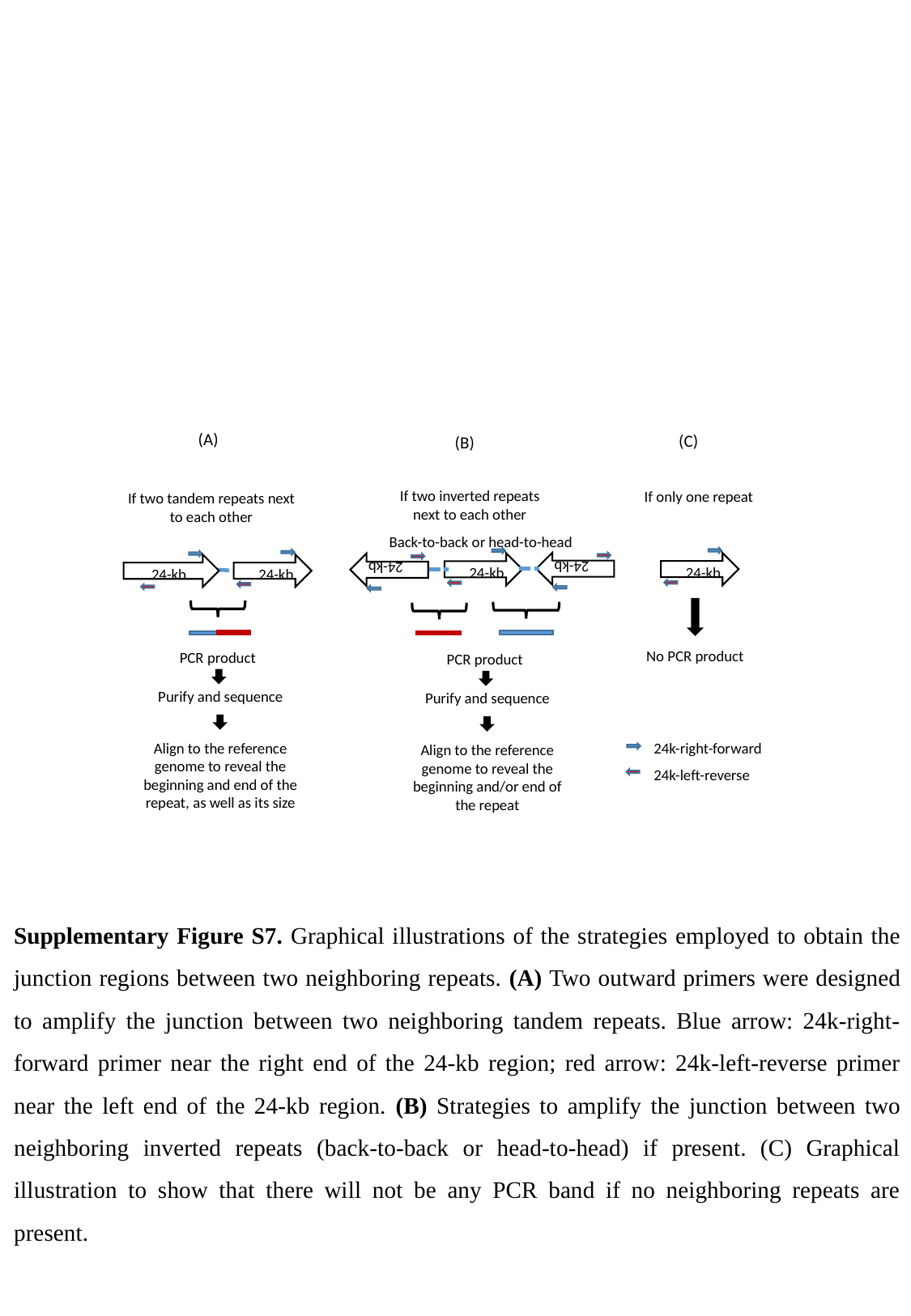

(A)
(C)
(B)
If two inverted repeats next to each other
If only one repeat
If two tandem repeats next to each other
Back-to-back or head-to-head
24-kb
24-kb
24-kb
24-kb
24-kb
24-kb
No PCR product
PCR product
PCR product
Purify and sequence
Purify and sequence
Align to the reference genome to reveal the beginning and end of the repeat, as well as its size
24k-right-forward
Align to the reference genome to reveal the beginning and/or end of the repeat
24k-left-reverse
Supplementary Figure S7. Graphical illustrations of the strategies employed to obtain the junction regions between two neighboring repeats. (A) Two outward primers were designed to amplify the junction between two neighboring tandem repeats. Blue arrow: 24k-right-forward primer near the right end of the 24-kb region; red arrow: 24k-left-reverse primer near the left end of the 24-kb region. (B) Strategies to amplify the junction between two neighboring inverted repeats (back-to-back or head-to-head) if present. (C) Graphical illustration to show that there will not be any PCR band if no neighboring repeats are present.

## Slide 8
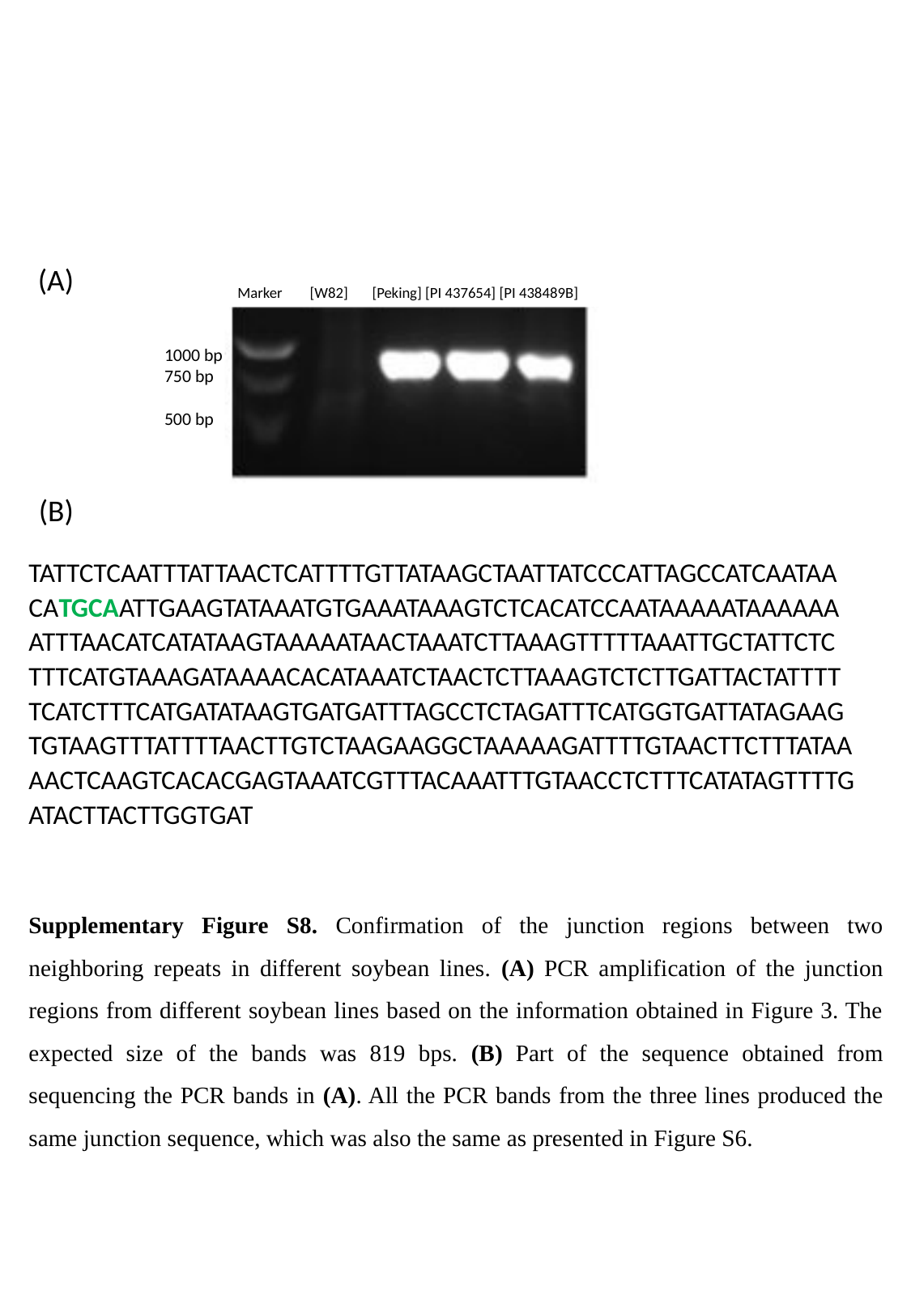

(A)
Marker [W82] [Peking] [PI 437654] [PI 438489B]
1000 bp
750 bp
500 bp
(B)
TATTCTCAATTTATTAACTCATTTTGTTATAAGCTAATTATCCCATTAGCCATCAATAA
CATGCAATTGAAGTATAAATGTGAAATAAAGTCTCACATCCAATAAAAATAAAAAA
ATTTAACATCATATAAGTAAAAATAACTAAATCTTAAAGTTTTTAAATTGCTATTCTC
TTTCATGTAAAGATAAAACACATAAATCTAACTCTTAAAGTCTCTTGATTACTATTTT
TCATCTTTCATGATATAAGTGATGATTTAGCCTCTAGATTTCATGGTGATTATAGAAG
TGTAAGTTTATTTTAACTTGTCTAAGAAGGCTAAAAAGATTTTGTAACTTCTTTATAA
AACTCAAGTCACACGAGTAAATCGTTTACAAATTTGTAACCTCTTTCATATAGTTTTG
ATACTTACTTGGTGAT
Supplementary Figure S8. Confirmation of the junction regions between two neighboring repeats in different soybean lines. (A) PCR amplification of the junction regions from different soybean lines based on the information obtained in Figure 3. The expected size of the bands was 819 bps. (B) Part of the sequence obtained from sequencing the PCR bands in (A). All the PCR bands from the three lines produced the same junction sequence, which was also the same as presented in Figure S6.

## Slide 9
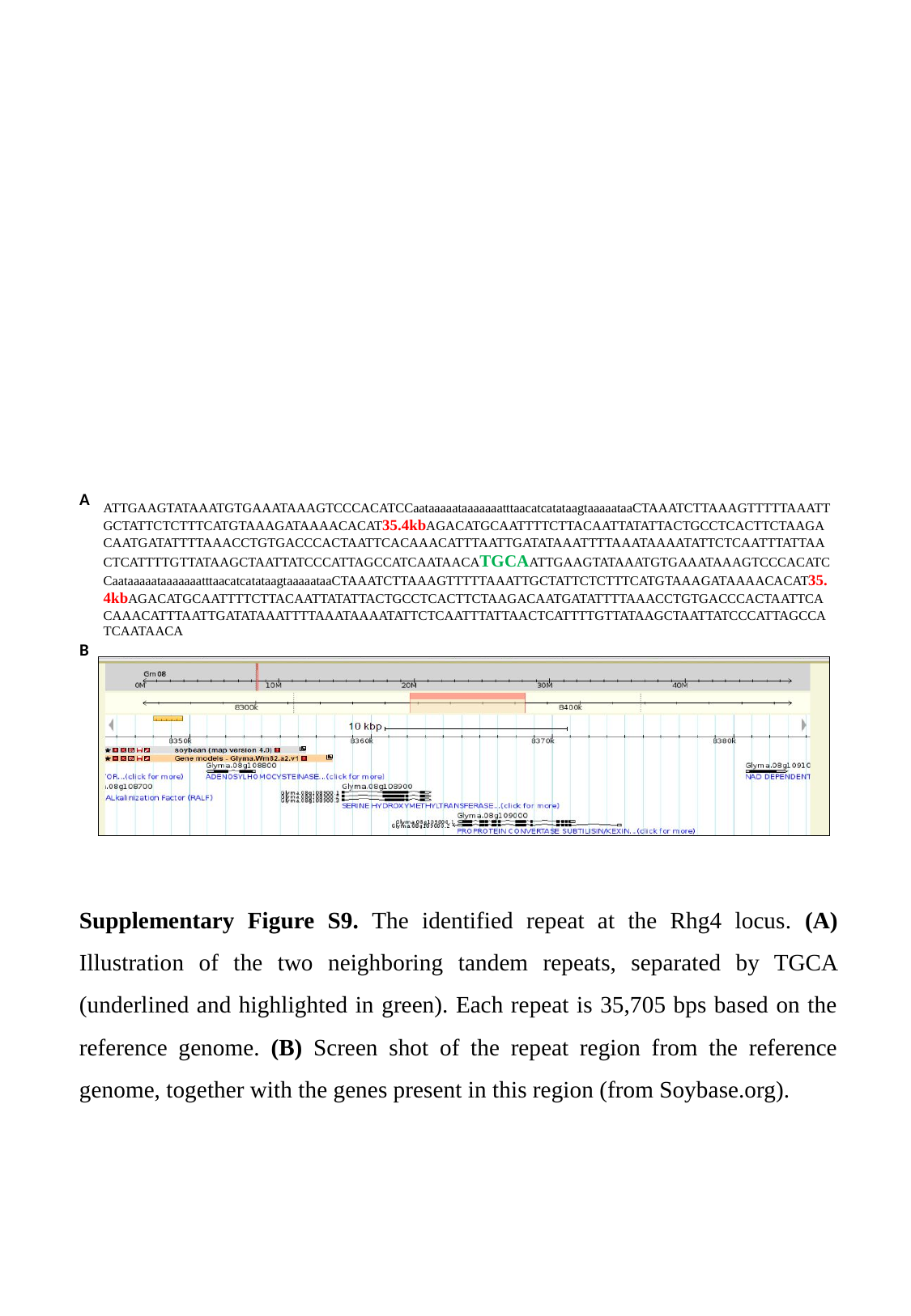

A
ATTGAAGTATAAATGTGAAATAAAGTCCCACATCCaataaaaataaaaaaatttaacatcatataagtaaaaataaCTAAATCTTAAAGTTTTTAAATTGCTATTCTCTTTCATGTAAAGATAAAACACAT35.4kbAGACATGCAATTTTCTTACAATTATATTACTGCCTCACTTCTAAGACAATGATATTTTAAACCTGTGACCCACTAATTCACAAACATTTAATTGATATAAATTTTAAATAAAATATTCTCAATTTATTAACTCATTTTGTTATAAGCTAATTATCCCATTAGCCATCAATAACATGCAATTGAAGTATAAATGTGAAATAAAGTCCCACATCCaataaaaataaaaaaatttaacatcatataagtaaaaataaCTAAATCTTAAAGTTTTTAAATTGCTATTCTCTTTCATGTAAAGATAAAACACAT35.4kbAGACATGCAATTTTCTTACAATTATATTACTGCCTCACTTCTAAGACAATGATATTTTAAACCTGTGACCCACTAATTCACAAACATTTAATTGATATAAATTTTAAATAAAATATTCTCAATTTATTAACTCATTTTGTTATAAGCTAATTATCCCATTAGCCATCAATAACA
B
Supplementary Figure S9. The identified repeat at the Rhg4 locus. (A) Illustration of the two neighboring tandem repeats, separated by TGCA (underlined and highlighted in green). Each repeat is 35,705 bps based on the reference genome. (B) Screen shot of the repeat region from the reference genome, together with the genes present in this region (from Soybase.org).
