# Supplementary material for: Whole‐genome re‐sequencing reveals the impact of the interaction of copy number variants of the rhg1 and Rhg4 genes on broad‐based resistance to soybean cyst nematode
Source: Plant Biotechnol J. 2019 Feb 20;17(8):1595–611. doi: 10.1111/pbi.13086 (PMC6662113; doi:10.1111/pbi.13086)
Supplement: Supplementary file 2 — Table S1 Statistics of DNA variant analysis for rhg1 from SCN‐resistant lines. Table S2 Statistics for DNA variant analysis of the rhg1 and Rhg4 loci from SCN‐resistant lines. Table S3 Primers used to study the Rhg4 duplication. Table S4 Summary of haplotype clusters, reaction to SCN races, CNV and type of rhg1 and Rhg4 resistance lines. Table S5 Requirement of rhg1 and Rhg4 copies in the presence and absence of GmSHMT08 promoter to confer SCN resistance. Table S6 Female index of soybean accessions used for gene expression analysis against five soybean cyst nematode populations: Race 1 (HG Type 2.5.7), Race 2 (HG Type 1.2.5.7), Race 3 (HG Type 0), Race 5 (HG Type 2.5.7) and Race 14 (HG Type 1.3.6.7). Table S7 Estimation of CNV using whole‐genome sequence and comparative genome hybridization in NAM population. [file PBI-17-1595-s001.docx]

**Supplementary Tables**

**Supplementary Table S1.** Statistics of DNA variant analysis for *rhg1* (24.8 kb) from SCN-resistant lines

| **Genotype** | **No** | **Variant Class** | | | | |
| --- | --- | --- | --- | --- | --- | --- |
|  |  | **Copy No.** | **SNPs** | **Insertion** | **Deletion** | **Total** |
| Maverick | HN030 | 9.43 | 187 | 30 | 32 | 249 |
| PI 597387 (Pana) | HN092 | 9.1 | 169 | 29 | 28 | 226 |
| PI 088788 | HN020 | 8.66 | 186 | 36 | 19 | 241 |
| PI 639740 | HN099 | 8.29 | 188 | 29 | 16 | 233 |
| PI 209332 | HN021 | 8.1 | 198 | 31 | 16 | 245 |
| PI 495017C | HN009 | 7.34 | 169 | 32 | 13 | 214 |
| PI 437655 | HN008 | 6.66 | 136 | 28 | 12 | 176 |
| PI 548316 | HN024 | 6.57 | 136 | 19 | 33 | 188 |
| PI 467312 | HN060 | 5.64 | 159 | 18 | 19 | 196 |
| PI 567519 | HN081 | 5.56 | 135 | 19 | 18 | 172 |
| PI 087631-1 | HN034 | 5.15 | 168 | 18 | 19 | 205 |
| PI 417091 | HN106 | 4.7 | 128 | 16 | 18 | 162 |
| PI 398610 | HN043 | 4.4 | 129 | 16 | 16 | 161 |
| PI 196175 | HN035 | 4.31 | 136 | 13 | 13 | 162 |
| PI 603154 | HN093 | 4.16 | 68 | 12 | 12 | 92 |
| PI 398593 | HN041 | 4.03 | 68 | 23 | 32 | 123 |
| PI 090763 | HN004 | 3.52 | 59 | 25 | 33 | 117 |
| PI 567305 | HN012 | 3.43 | 96 | 16 | 19 | 131 |
| PI 437679 | HN055 | 3.26 | 78 | 13 | 8 | 99 |
| PI 437654 | HN015 | 3.21 | 58 | 12 | 7 | 77 |
| PI 567516C | HN025 | 3.16 | 36 | 32 | 16 | 84 |
| PI 548402B | HN002 | 3.15 | 199 | 33 | 12 | 244 |
| PI 437690 | HN018 | 3.15 | 125 | 19 | 13 | 157 |
| S05-11482 | HN013 | 3.16 | 136 | 18 | 16 | 170 |
| PI 567230 | HN075 | 3.06 | 69 | 19 | 18 | 106 |
| PI 089772 | HN003 | 3.05 | 48 | 18 | 16 | 82 |
| PI 612611 | HN026 | 3.04 | 68 | 16 | 8 | 92 |
| PI 567336B | HN076 | 2.98 | 36 | 9 | 6 | 51 |
| PI 507354 | HN011 | 2.95 | 69 | 8 | 7 | 84 |
| PI 548402 (Peking) | HN019 | 2.93 | 58 | 7 | 11 | 76 |
| PI 603176A | HN096 | 2.88 | 63 | 16 | 16 | 95 |
| PI 407788A | HN006 | 2.85 | 28 | 12 | 12 | 52 |
| PI 437725 | HN017 | 2.84 | 56 | 13 | 14 | 83 |
| PI 468915 | HN010 | 2.75 | 48 | 17 | 8 | 73 |
| PI 424298 | HN007 | 2.74 | 35 | 16 | 7 | 58 |
| PI 658519 | HN101 | 2.67 | 26 | 18 | 6 | 50 |
| PI 424608A | HN023 | 2.64 | 63 | 16 | 7 | 86 |
| PI 603497 | HN097 | 2.64 | 54 | 8 | 6 | 68 |
| PI 567387 | HN016 | 2.6 | 28 | 6 | 8 | 42 |
| PI 404166 | HN005 | 2.54 | 45 | 7 | 11 | 63 |
| S10-11227 | HN027 | 2.41 | 45 | 9 | 12 | 66 |
| PI 404198B | HN022 | 1.91 | 96 | 11 | 12 | 119 |
| Average. |  | 4.23 | 97 | 18 | 14 | 130 |

**Supplementary Table S2.** Statistics for DNA variant analysis of the *rhg1* and *Rhg4* loci from SCN-resistant lines.

| **Genotype** | **No** | ***rhg1* Variant Class** | | | | |  | | ***Rhg4* Variant Class** | | | | |
| --- | --- | --- | --- | --- | --- | --- | --- | --- | --- | --- | --- | --- | --- |
|  |  | **CNV** | **SNPs** | **Insertion** | **Deletion** | **Total** |  | **CNV** | | **SNPs** | **Insertion** | **Deletion** | **Total** |
| PI 468915 | HN010 | 2.75 | 48 | 17 | 8 | 73 |  | 4.34 | | 36 | 8 | 7 | 51 |
| PI 437654 | HN015 | 3.27 | 58 | 12 | 7 | 77 |  | 4.3 | | 35 | 9 | 11 | 55 |
| PI 404198B | HN022 | 1.91 | 96 | 11 | 12 | 119 |  | 3.37 | | 32 | 11 | 9 | 52 |
| PI 437690 | HN018 | 3.15 | 125 | 19 | 13 | 157 |  | 3.27 | | 29 | 12 | 8 | 49 |
| PI 090763 | HN004 | 3.52 | 59 | 25 | 33 | 117 |  | 2.82 | | 28 | 12 | 9 | 49 |
| PI 089772 | HN003 | 3.50 | 48 | 18 | 16 | 82 |  | 2.41 | | 26 | 13 | 13 | 52 |
| PI 437679 | HN055 | 3.26 | 78 | 13 | 8 | 99 |  | 2.24 | | 36 | 8 | 9 | 53 |
| PI 404166 | HN005 | 2.54 | 45 | 7 | 11 | 63 |  | 2.21 | | 29 | 10 | 9 | 48 |
| PI 548402 | HN019 | 3.2 | 58 | 7 | 11 | 76 |  | 2.29 | | 27 | 10 | 12 | 49 |
| PI 437725 | HN017 | 2.84 | 56 | 13 | 14 | 83 |  | 2.80 | | 26 | 10 | 12 | 48 |
| Ave. |  | 2.94 | 79 | 16 | 13 | 108 |  | 2.85 | | 31 | 10 | 10 | 51 |

**Supplementary Table S3. Primers used to study the *Rhg4* duplication**

| **Primer name** | **Primer sequence (5' to 3')** | **Note** |
| --- | --- | --- |
| 16k-forward | CCGTCACAAAAGACTCGGTTTG | To amplify a ~16-kb sequence within the Rhg4 repeat |
| 16k-reverse | TTGACCACACTCTCATCATCTC |  |
| 20k-forward | GACAAATTCTGTGGGTTCATTTG | To amplify a ~20-kb sequence within the Rhg4 repeat |
| 20k-reverse | GGCATTGCAATTTGCAACCTTG |  |
| 24k-forward | GGGATTGAGTGTCATGCGTTAG | To amplify a ~24-kb sequence within the Rhg4 repeat |
| 24k-reverse | GGGTTCCAATCACGCTATTAGTG |  |
| 24k-right-forward | CAGATTGGGACTTGAAGGTTAAC | To amplify the junction region between two neighobring repeats |
| 24k-left-reverse | GTAGGTGTGTTAAACGTGTTCGAC |  |
| Junction113-forward | CCTGTGACCCACTAATTCACAAAC | To amplify a short region between two neighobring repeats |
| Junction704-reverse | CATCTCGTGCTAGGTTTGGTTG |  |

**Supplementary Table S4:** Summary of Haplotype clusters, reaction to SCN races, CNV and type of *rhg-1* and *Rhg-4* resistance lines. **(A)** PI88788 and “Cloud” type resistance. **(B)** “Peking” type resistance. **(C)** Susceptible lines (W82-type).

**(A)**

|  | Seq id | PI # (name) | Line  Type | Female Index | | | | | *rhg1*-type | *Rhg4*-type | CNV | |
| --- | --- | --- | --- | --- | --- | --- | --- | --- | --- | --- | --- | --- |
|  |  |  |  | PA1 | PA2 | PA3 | PA5 | PA14 |  |  | rhg1 | Rhg4 |
| Group-4 (Rhg1-b + Rhg4-b) | HN020 | PI088788 | Landrace | 26 | 29 | 2 | 46 | 7 | PI88788 | WT | 8.7 | 1.0 |
|  | HN021 | PI209332 | Landrace | 44 | 32 | 2 | 76 | 6 | PI88788 | WT | 8.1 | 1.0 |
|  | HN043 | PI398610 | Elite | 66 | 73 | 43 | 76 | 68 | PI88788 | WT | 4.4 | 1.0 |
|  | HN035 | PI196175 | Elite | 69 | 73 | 55 | 87 | 69 | PI88788 | WT | 4.3 | 1.0 |
|  | HN041 | PI398593 | Elite | 70 | 82 | 51 | 82 | 68 | PI88788 | WT | 4.3 | 1.0 |
|  | HN093 | PI603154 | Elite | 86 | 74 | 45 | 78 | 86 | PI88788 | WT | 4.2 | 1.0 |
|  | HN026 | PI612611 | Landrace | 59 | 45 | 31 | 13 | 56 | PI88788 | WT | 3.4 | 1.0 |
|  | HN096 | PI603176A | Elite | 74 | 75 | 50 | 87 | 89 | PI88788 | WT | 2.9 | 1.0 |
| Group-5 (Rhg1-b1 + Rhg4-b) | HN030 | Maverick | Elite | 57 | 51 | 2 | 71 | 3 | Cloud | WT | 9.4 | 1.0 |
|  | HN092 | PI597387 | Elite | 41 | 43 | 1 | 66 | 3 | Cloud | WT | 9.1 | 1.0 |
|  | HN099 | PI639740 | Elite | 41 | 50 | 3 | 58 | 26 | Cloud | WT | 8.3 | 1.0 |
|  | HN009 | PI495017C | Landrace | 37 | 17 | 3 | 57 | 9 | Cloud | WT | 7.3 | 1.0 |
|  | HN008 | PI437655 | Landrace | ** | 30 | ** | 19 | . | Cloud | WT | 6.7 | 1.0 |
|  | HN024 | PI548316 | Landrace | 63 | 40 | 4 | 68 | 20 | Cloud | WT | 6.6 | 1.0 |
|  | HN060 | PI467312 | Landrace | 52 | 41 | 2 | 37 | 15 | Cloud | WT | 5.6 | 1.0 |
|  | HN081 | PI567519 | Elite | 68 | 73 | 68 | 41 | 84 | Cloud | WT | 5.6 | 1.0 |
|  | HN034 | PI087631-1 | Landrace | 34 | 38 | 1 | 55 | 13 | Cloud | WT | 5.2 | 1.0 |
|  | HN106 | PI417091 | Landrace | 74 | 27 | 5 | 43 | 17 | Cloud | WT | 4.7 | 1.0 |
|  | HN039 | PI366121 | Wild | 29 | 46 | 71 | 52 | 49 | Cloud | WT |  |  |

**(B)**

|  | Seq id | PI # (name) | Line  Type | | Female Index | | | | | | | | | *rhg1*-type | *Rhg4*-type | | CNV | | |  |
| --- | --- | --- | --- | --- | --- | --- | --- | --- | --- | --- | --- | --- | --- | --- | --- | --- | --- | --- | --- | --- |
|  |  |  |  |  | PA1 | | PA2 | | PA3 | | PA5 | | PA14 |  |  |  | rhg1 | Rhg4 | |  |
| Group-1 (Rhg1-a + Rhg4-a) | HN004 | PI090763 | Landrace | 2 | | 4 | | 2 | | 2 | | 3 | | Peking | | Peking | 3.5 | | 2.8 | |
|  | HN003 | PI089772 | Landrace | 1 | | 12 | | 1 | | 1 | | 31 | | Peking | | Peking | 3.5 | | 2.4 | |
|  | HN055 | PI437679 | Landrace | 1 | | 19 | | 1 | | 2 | | 23 | | Peking | | Peking | 3.3 | | 2.2 | |
|  | HN002 | PI548402 | Landrace | 1 | | 53 | | 1 | | 12 | | 67 | | Peking | | Peking | 3.2 | | 2.3 | |
|  | HN019 | PI548402 | Landrace | 3 | | 38 | | 2 | | 4 | | 40 | | Peking | | Peking | 3.2 | | 2.3 | |
|  | HN018 | PI437690 | Landrace | 1 | | 59 | | 2 | | 4 | | 67 | | Peking | | Peking | 3.2 | | 3.3 | |
|  | HN011 | PI507354 | Landrace | 1 | | 58 | | 2 | | 6 | | 90 | | Peking | | Peking | 3.0 | | 1.1 | |
|  | Forrest |  | Landrace | 3 | | 32 | | 3 | | 5 | | 56 | | Peking | | Peking | 2.9 | | 1.1 | |
|  | HN017 | PI437725 | Landrace | 0 | | 58 | | 2 | | 3 | | 70 | | Peking | | Peking | 2.8 | | 2.8 | |
|  | HN010 | PI468915 | Landrace | 1 | | 40 | | 1 | | 5 | | 80 | | Peking | | Peking | 2.8 | | 4.3 | |
|  | HN101 | PI658519 | Elite | 15 | | 40 | | 1 | | 2 | | 69 | | Peking | | Peking | 2.7 | | 1.0 | |
|  | HN005 | PI404166 | Landrace | 1 | | 25 | | 1 | | 1 | | 34 | | Peking | | Peking | 2.5 | | 2.2 | |
|  | HN022 | PI404198B | Landrace | 1 | | 51 | | 1 | | 12 | | 53 | | Peking | | Peking | 1.9 | | 3.4 | |
| Group-2 (Rhg1-a + Rhg4-c) | HN015 | PI437654 | Landrace | 1 | | 2 | | 1 | | 2 | | 3 | | Peking | | Peking | 3.3 | | 4.3 | |
| Group-3 (Rhg1-a + Rhg4-b) | HN075 | PI567230 | Landrace | 55 | | 64 | | 44 | | ** | | ** | | Peking | | WT | 3.6 | | 1.0 | |
|  | HN012 | PI567305 | Landrace | 30 | | 5 | | 22 | | 1 | | 38 | | Peking | | WT | 3.4 | | 1.0 | |
|  | HN025 | PI567516C | Landrace | 27 | | 18 | | 15 | | 2 | | 15 | | Peking | | WT | 3.2 | | 1.0 | |
|  | HN076 | PI567336B | Landrace | 27 | | 14 | | 34 | | 1 | | 52 | | Peking | | WT | 3.0 | | 1.0 | |
|  | HN006 | PI407788A | Elite | 80 | | 48 | | 64 | | 5 | | 80 | | Peking | | WT | 2.9 | | 1.0 | |
|  | HN007 | PI424298 | Landrace | 36 | | 70 | | 68 | | 11 | | 91 | | Peking | | WT | 2.7 | | 1.0 | |
|  | HN097 | PI603497 | Landrace | 73 | | 63 | | 60 | | 5 | | 76 | | Peking | | WT | 2.6 | | 1.0 | |
|  | HN023 | PI424608A | Landrace | 62 | | 50 | | 54 | | 5 | | 80 | | Peking | | WT | 2.6 | | 1.0 | |
|  | HN016 | PI567387 | Landrace | 54 | | 21 | | 59 | | 4 | | 73 | | Peking | | WT | 2.6 | | 1.0 | |
|  | HN027 | S10-11227 | Elite | 62 | | 13 | | 16 | | 3 | | 9 | | Peking | | WT | 2.5 | | 1.0 | |

**(C)**

| Group-6 (Rhg1-c + Rhg4-b) | HN107 | PI417015 | Elite | 74 | 83 | 65 | 80 | 103 | WT | WT |  |  |
| --- | --- | --- | --- | --- | --- | --- | --- | --- | --- | --- | --- | --- |
|  | HN105 | PI438471 | Elite | 55 | 78 | 80 | 122 | 104 | WT | WT |  |  |
|  | HN104 | FC 31721 | Landrace | 75 | 76 | 70 | 70 | 74 | WT | WT |  |  |
|  | HN103 | V71-370 | Elite | 72 | 85 | 62 | 89 | 113 | WT | WT |  |  |
|  | HN102 | S07-5049 | Elite | 65 | 77 | 62 | 82 | 63 | WT | WT |  |  |
|  | HN100 | PI647086 | Elite | 83 | 94 | 68 | 97 | 92 | WT | WT |  |  |
|  | HN098 | PI605869A | Elite | 79 | 85 | 71 | 93 | 95 | WT | WT |  |  |
|  | HN095 | PI603175 | Elite | 86 | 82 | 74 | 101 | 95 | WT | WT |  |  |
|  | HN094 | PI603170 | Elite | 77 | 85 | 51 | 96 | 91 | WT | WT |  |  |
|  | HN091 | PI594599 | Elite | 75 | 89 | 75 | 116 | 94 | WT | WT |  |  |
|  | HN090 | PI594512A | Elite | 82 | 94 | 68 | 91 | 85 | WT | WT |  |  |
|  | HN089 | PI594012 | Elite | 72 | 87 | 78 | 88 | 97 | WT | WT |  |  |
|  | HN088 | PI593258 | Elite | 68 | 69 | 54 | 73 | 93 | WT | WT | 1.0 | 1.0 |
|  | HN087 | PI591539 | Elite | 72 | 77 | 65 | 79 | 84 | WT | WT |  |  |
|  | HN086 | PI567731 | Elite | 87 | 71 | 64 | 80 | 86 | WT | WT |  |  |
|  | HN085 | PI567719 | Elite | 73 | 79 | 52 | 93 | 92 | WT | WT |  |  |
|  | HN084 | PI567690 | Elite | 85 | 77 | 76 | 86 | 91 | WT | WT |  |  |
|  | HN083 | PI567651 | Elite | 82 | 89 | 78 | 88 | 85 | WT | WT |  |  |
|  | HN082 | PI567611 | Elite | 73 | 80 | 62 | 77 | 80 | WT | WT |  |  |
|  | HN080 | PI567383 | Landrace | 70 | 145 | 40 | 67 | 69 | WT | WT |  |  |
|  | HN079 | PI567357 | Landrace | 76 | 78 | 64 | 92 | 89 | WT | WT |  |  |
|  | HN078 | PI567354 | Landrace | 71 | 84 | 57 | 77 | 69 | WT | WT |  |  |
|  | HN077 | PI567343 | Landrace | 61 | 80 | 58 | 65 | ** | WT | WT |  |  |
|  | HN074 | PI561271 | Elite | 86 | 70 | 81 | 68 | 67 | WT | WT |  |  |
|  | HN073 | PI552538 | Elite | 74 | 78 | 69 | 86 | 90 | WT | WT |  |  |
|  | HN072 | PI549031 | Landrace | 64 | 59 | 41 | 27 | 65 | WT | WT |  |  |
|  | HN071 | PI548657 | Elite | 73 | 86 | 72 | 90 | 90 | WT | WT |  |  |
|  | HN070 | PI548511 | Elite | 66 | 65 | 74 | 94 | 101 | WT | WT |  |  |
|  | HN069 | PI548415 | Landrace | 66 | 54 | 46 | 71 | 60 | WT | WT |  |  |
|  | HN068 | PI548349 | Landrace | 41 | 48 | 18 | 52 | 31 | WT | WT | 1.1 | 1.0 |
|  | HN067 | PI548317 | Landrace | 25 | 28 | 30 | 27 | 33 | WT | WT | 1.2 | 1.0 |
|  | HN065 | PI542044 | Elite | 86 | 85 | 69 | 89 | 93 | WT | WT |  |  |
|  | HN064 | PI518751 | Elite | 59 | 85 | 52 | 84 | 89 | WT | WT |  |  |
|  | HN063 | PI483463 | Wild | . | . | . | . | ** | WT | WT |  |  |
|  | HN062 | PI475783B | Elite | 83 | 99 | 77 | 107 | 90 | WT | WT |  |  |
|  | HN061 | PI471938 | Elite | 71 | 94 | 66 | 84 | 86 | WT | WT |  |  |
|  | HN059 | PI464920B | Elite | 64 | 70 | 46 | 65 | 78 | WT | WT |  |  |
|  | HN058 | PI458515 | Landrace | 67 | 62 | 52 | 61 | 66 | WT | WT |  |  |
|  | HN057 | PI438258 | Elite | 96 | 96 | 61 | 99 | 111 | WT | WT |  |  |
|  | HN056 | PI437863A | Elite | 74 | 76 | 67 | 78 | 75 | WT | WT |  |  |
|  | HN054 | PI437169B | Elite | 74 | 78 | 53 | 82 | 103 | WT | WT |  |  |
|  | HN053 | PI424088 | Wild | 32 | 22 | 63 | . | . | WT | WT |  |  |
|  | HN052 | PI424079 | Wild | . | 21 | . | . | . | WT | WT |  |  |
|  | HN051 | PI424078 | Landrace | 67 | 86 | ** | ** | ** | WT | WT |  |  |
|  | HN050 | PI416937 | Elite | 80 | 87 | 76 | 80 | 94 | WT | WT |  |  |
|  | HN049 | PI408105A | Elite | 78 | 85 | 71 | 104 | 94 | WT | WT |  |  |
|  | HN048 | PI407965 | Elite | 81 | 85 | 65 | 78 | 80 | WT | WT |  |  |
|  | HN047 | PI407729 | Landrace | 28 | 13 | 10 | 12 | 13 | WT | WT | 1.2 | 1.0 |
|  | HN046 | PI407184 | Wild | 49 | 56 | 60 | 70 | 48 | WT | WT |  |  |
|  | HN045 | PI407162 | Wild | 53 | 50 | 66 | 71 | ** | WT | WT |  |  |
|  | HN044 | PI398614 | Elite | 63 | 85 | 61 | 86 | 84 | WT | WT |  |  |
|  | HN042 | PI398595 | Elite | 74 | 87 | 80 | 76 | 79 | WT | WT |  |  |
|  | HN040 | PI378702 | Wild | 69 | ** | 57 | 70 | 51 | WT | WT |  |  |
|  | HN038 | PI248515 | Elite | 82 | 81 | 74 | 106 | 99 | WT | WT |  |  |
|  | HN037 | PI200508 | Elite | 84 | 95 | 90 | 100 | 99 | WT | WT |  |  |
|  | HN033 | PI087617 | Elite | 83 | 88 | 66 | 103 | 105 | WT | WT |  |  |
|  | HN032 | PI086006 | Landrace | 86 | 104 | 86 | 101 | 93 | WT | WT |  |  |
|  | HN031 | PI079691-4 | Landrace | 87 | 84 | 54 | 75 | 82 | WT | WT |  |  |
|  | HN029 | IA3023 | Elite | 64 | 73 | 58 | 70 | 85 | WT | WT |  |  |
|  | HN014 | PI548667 | Elite | 67 | 83 | 74 | 68 | 88 | WT | WT | 1.1 | 1.0 |
|  | HN028 | Holladay | Elite | 85 | 97 | 77 | 90 | 103 | WT | WT |  |  |
|  | HN066 | PI547862 | Elite | 70 | 78 | 59 | 77 | 106 | WT | WT |  |  |
|  | W82 | Reference | Elite | 82 | 81 | 78 | 75 | 85 | WT | WT | 1.0 | 1.0 |
|  | HN001 | PI518664 | Elite | 79 | 76 | 54 | 83 | 94 | WT | WT | 1.1 | 1.0 |

**NOTE:** FI was calculated as follow: Female Index (FI) values = (mean number of females on test cultivar) ÷ (mean number of females on control) x 100.

**Supplementary Table S5:** Requirement of *rhg1* and *Rgh4* copies in presence and absence of *GmSHMT08* promoter to confer SCN resistance.

| Promoter-> | **PI88788-type** | | **‘Cloud’-type** | | **‘Peking’-type** | |
| --- | --- | --- | --- | --- | --- | --- |
|  | SHMT- | SHMT+ | SHMT- | SHMT+ | SHMT- | SHMT+ |
| **Rhg1 (CNV)** | **8.1** | **3.4** | **7.34** | **4.7** | **2.47** | **1.91** |
| Rhg4 (CNV) | 1 | 1.1 | 1 | 1 | 1 | 3.37 |

**Supplementary Table S6.** Female indexes of soybean accessions used for gene expression analysis against five soybean cyst nematode populations: Race 1 (HG Type 2.5.7), Race 2 (HG Type 1.2.5.7), Race 3 (HG Type 0), Race 5 (HG Type 2.5.7), and Race 14 (HG Type 1.3.6.7).

|  | **Accession** | **Seq Id** | **Haplotype** | **SCN Female Index Value*** | | | | |
| --- | --- | --- | --- | --- | --- | --- | --- | --- |
|  |  |  |  | **Race 1** | **Race 2** | **Race 3** | **Race 5** | **Race 14** |
| 1 | PI 437654 | HN015 | *rhg1-a* + *Rhg4-c* | 1 | 2 | 1 | 2 | 3 |
| 2 | PI 090763 | HN004 | *rhg1-a* + *Rhg4-a* | 2 | 4 | 2 | 2 | 32 |
| 3 | Peking | HN019 | *rhg1-a* + *Rhg4-a* | 3 | 38 | 2 | 4 | 40 |
| 4 | PI 88788 | HN020 | *rhg1-b* + *Rhg4-b* | 26 | 29 | 2 | 46 | 7 |
| 5 | Essex | HN014 | *rhg1-c* + *Rhg4-b* | 67 | 83 | 74 | 68 | 88 |

*SCN Female Index rating system: FI=0-9, resistant; 10-29, moderate resistance; 30-59 moderate susceptibility; >60, susceptibility.

**Supplementary Table S7:** Estimation of CNV using whole genome sequence and comparative genome hybridization in NAM population. The WGRS and CHG data was accessed from Stupar Lab, University of Minnesota, MN. (<http://stuparlabcnv.cfans.umn.edu:8080/>).

|  |  | ***rhg1*** | | ***Rhg4*** | |
| --- | --- | --- | --- | --- | --- |
| **Parent Name** | **Type** | **CGH** | **WGRS** | **CGH** | **WGRS** |
| IA3023 | Parent | 1.03 | 0.81 | 1.04 | 0.77 |
| TN05-3027 | High Yieldling Lines | 1.01 | 0.79 | 0.95 | 1.03 |
| 4J105-3-4 | High Yieldling Lines | 5.97 | 8.47 | 0.95 | 0.94 |
| 5M20-2-5-2 | High Yieldling Lines | 1.01 | 0.80 | 0.97 | 0.94 |
| CL0J095-4-6 | High Yieldling Lines | 5.86 | 7.98 | 0.84 | 0.76 |
| CL0J173-6-8 | High Yieldling Lines | 1.03 | 0.88 | 0.96 | 1.01 |
| HS6-3976 | High Yieldling Lines | 1.02 | 0.88 | 0.93 | 0.67 |
| Prohio | High Yieldling Lines | 1.08 | 0.71 | 0.97 | 0.80 |
| LD00-3309 | High Yieldling Lines | 6.42 | 8.15 | 0.93 | 0.79 |
| LD01-5907 | High Yieldling Lines | 2.19 | 2.33 | 0.96 | 0.88 |
| LD02-4485 | High Yieldling Lines | 6.79 | 7.86 | 1.00 | 0.79 |
| LD02-9050 | High Yieldling Lines | 6.58 | 7.95 | 0.96 | 0.94 |
| Magellan | High Yieldling Lines | 0.99 | 0.84 | 0.97 | 1.07 |
| Maverick | High Yieldling Lines | 6.40 | 8.78 | 1.03 | 0.90 |
| S06-13640 | High Yieldling Lines | 1.02 | 0.80 | 1.02 | 0.52 |
| NE3001 | High Yieldling Lines | 0.98 | 0.89 | 1.00 | 0.89 |
| Skylla | High Yieldling Lines | 1.06 | 0.69 | 1.06 | 0.72 |
| U03-100612 | High Yieldling Lines | 1.02 | 0.76 | 1.01 | 0.82 |
| LG03-2979 | Diverse Ancestry | 1.07 | 0.84 | 0.98 | 0.94 |
| LG03-3191 | Diverse Ancestry | 1.07 | 0.94 | 0.98 | 0.78 |
| LG04-4717 | Diverse Ancestry | 1.09 | 0.84 | 0.93 | 0.94 |
| LG05-4292 | Diverse Ancestry | 5.54 | 7.22 | 0.98 | 0.90 |
| LG05-4317 | Diverse Ancestry | 0.96 | 0.87 | 0.98 | 0.87 |
| LG05-4464 | Diverse Ancestry | 1.07 | 0.79 | 0.95 | 0.90 |
| LG05-4832 | Diverse Ancestry | 1.05 | 0.77 | 0.91 | 0.96 |
| LG90-2550 | Diverse Ancestry | 1.09 | 0.99 | 0.94 | 0.89 |
| LG92-1255 | Diverse Ancestry | 1.03 | 0.84 | 0.99 | 0.96 |
| LG94-1128 | Diverse Ancestry | 1.01 | 1.09 | 0.95 | 0.70 |
| LG94-1906 | Diverse Ancestry | 1.06 | 0.78 | 0.99 | 0.98 |
| LG97-7012 | Diverse Ancestry | 1.06 | 0.84 | 1.00 | 0.58 |
| LG98-1605 | Diverse Ancestry | 1.04 | 0.78 | 0.93 | 0.80 |
| LG00-3372 | Diverse Ancestry | 1.04 | 0.78 | 0.98 | 0.70 |
| LG04-6000 | Diverse Ancestry | 0.93 | 1.01 | 0.97 | 0.66 |
| PI398881 | High Yield + Drought | 1.07 | 1.02 | 0.92 | 0.93 |
| PI427136 | High Yield + Drought | 1.01 | 0.86 | 0.90 | 0.83 |
| PI437169B | High Yield + Drought | 0.93 | 0.79 | 1.01 | 1.07 |
| PI507681B | High Yield + Drought | 1.05 | 0.77 | 0.91 | 0.76 |
| PI518751 | High Yield + Drought | 1.05 | 1.03 | 1.04 | 0.97 |
| PI561370 | High Yield + Drought | 1.04 | 0.76 | 1.07 | 0.96 |
| PI404188A | High Yield + Drought | 1.04 | 0.86 | 1.06 | 0.78 |
| PI574486 | High Yield + Drought | 1.05 | 0.86 | 1.09 | 0.83 |
